# Genome‐wide analysis of colorectal cancer based on gene‐based somatic copy number alterations during neoplastic progression within the same tumor

**DOI:** 10.1002/cam4.5117

**Published:** 2022-08-03

**Authors:** Shun Yamada, Mitsumasa Osakabe, Noriyuki Uesugi, Naoki Yanagawa, Takayuki Matsumoto, Hiromu Suzuki, Tamotsu Sugai

**Affiliations:** ^1^ Department of Molecular Diagnostic Pathology, School of Medicine Iwate Medical University Yahaba Japan; ^2^ Division of Gastroenterology, Department of Internal Medicine Iwate Medical University Yahaba Japan; ^3^ Department of Molecular Biology Sapporo Medical University Sapporo Japan

**Keywords:** colorectal cancer, gene somatic copy number alteration, mesothelin, SNP array

## Abstract

**Background:**

The objective of this study was to elucidate the association between neoplastic progression and somatic copy number alterations (SCNAs) occurring within the same colorectal cancer (CRC) tumor.

**Methods:**

We investigated SCNAs to identify the progression from a high‐grade intramucosal lesion (HGIL) to an invasive front lesion (IFL), via an invasive submucosal lesion (ISL), within the same tumor using a crypt isolation method combined with a SNP array. Immunohistochemistry was also performed.

**Results:**

We identified 51 amplified genes that potentially promote progression from HGIL to ISL and 6 amplified genes involved in the progression from ISL to IFL. Of the 51 genes involved in HGIL to ISL progression, *TORC1*, *MSLN*, and *STUB1*, which are closely associated with CRC, were identified as candidate markers of submucosal invasion. However, no candidate genes were identified among the six genes associated with ISL to IFL progression. In addition, the number of total SCNAs and the number of gains were correlated with cancer progression (from HGIL to IFL). Finally, immunohistochemistry revealed higher expression of *TORC1*, *MSLN*, and *STUB1* in ISL than in HGIL.

**Conclusions:**

These results suggest that specific SCNAs are required for acquisition of invasive ability in CRC, and the affected genes are potential markers of invasion.

## Graphical abstract

We identified 51 amplified genes that potentially progression from a high‐grade intramucosal lesion (HGIL) to an invasive submucosal lesion (ISL) and 6 amplified genes involved in the progression from ISL to an invasive front lesion (IFL) within the same colorectal cancer tumor.

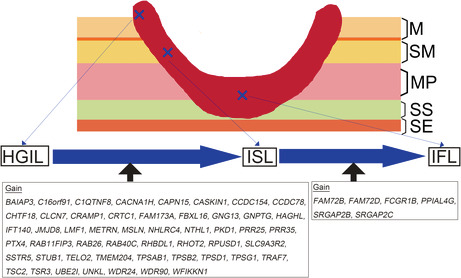

## 1 INTRODUCTION

Colorectal tumorigenesis is a multistep process involving accumulation of genetic alterations during progression from adenoma to invasive carcinoma via an intermediate stage (invasive submucosal lesion [ISL]).^1^, ^2^, ^3^, ^4^ The model of Vogelstein et al. explains colorectal carcinogenesis based on the adenoma–carcinoma sequence involving several molecular features.^1^, ^2^, ^3^, ^4^ According to this hypothesis, colorectal tumors result from activation of oncogenes via mutations coupled with inactivation of tumor suppressor genes.^1^ Although the Vogelstein model explained how chromosomal instability occurs finally through inactivation of *TP53* function, his model has not been successful in demonstrating how chromosomal instability causes enhanced invasive activity by identifying responsible genes. Genetic alterations closely associated with colorectal carcinogenesis include gene mutations, DNA methylation, dysregulated microRNA expression, and somatic copy number alterations (SCNAs).^5^, ^6^, ^7^ Although a combination rather than one of these molecular alteration types contributes to development of colorectal cancer (CRC), SCNAs are attracting attention as a molecular mechanism of tumor progression.^5^, ^6^, ^7^ In fact, a recent study showed that SCNAs accumulate during tumor progression and play a crucial role in neoplastic invasion through the mucosa.^8^ Although the total number of changes, rather than their chronological order of appearance, may determine the biological properties of a tumor, SCNAs as well as genetic mutations often occur in a specific sequence.^9^

Genome‐wide comprehensive profiling of SCNAs in CRC has been performed to elucidate the extent and distribution of SCNAs in the CRC genome.^10^, ^11^, ^12^ Despite these advances, the main challenge is distinguishing the alterations playing causative roles (drivers) from the random alterations that accumulate during colorectal carcinogenesis (passengers).^3^, ^5^ Integrating these identified SCNA regions with functional knowledge of affected genes would help determine the importance of different genes in CRC initiation and progression.^10^, ^11^, ^12^ For uncharacterized genes, the extent of their SCNA alterations is often used to judge their relevance to cancer.

Although many studies have demonstrated SCNAs in specific genes and their diagnostic/prognostic significance in different lesion types (adenoma and invasive CRC),^9^, ^10^, ^12^ a comprehensive study of the involvement of SCNAs in the progression from an intramucosal lesion with high‐grade dysplasia or intramucosal cancer (IMC) to invasive CRC, via an ISL, within the same tumor has not been performed so far. In this study, we systematically analyzed SCNAs and their affected genes during CRC progression, specifically focusing on progression within the same tumor; this novel model is important for evaluating progression from IMC to invasive CRC.

## 2 PATIENTS

Twenty‐three patients were obtained from 887 CRC patients who were consecutively operated on at Iwate Medical University between 2018 and 2021. The tumors from these 23 patients consisted of three components within the same tumor: high‐grade intramucosal lesion (HGIL; IMC or high‐grade adenoma), ISL, and invasive front lesion (IFL). Although this model is important to evaluate tumor progression within the same tumor, lesions involving all three components within the same tumor are very rare in routine pathological diagnosis. The existence of this model was confirmed by histological analysis. Clinicopathological findings, including tumor location, macroscopic type, histological classification, lymphatic invasion, venous invasion, and tumor stage, were determined according to the Classification of the Japanese Society for Cancer of the Colon and Rectum.^13^ In addition, the histopathological diagnosis was performed using concise concrete description with references.^13^ Histological type examined in this study was intestinal‐type cancer which shows glandular differentiation. The detailed clinicopathological findings are shown in Table 1.

**TABLE 1:** Clinicopathological findings

	This study
Total cases	23
Sex	
Male	15 (65.2)
Female	8 (34.8)
Age, year, median [range]	69 [42–86]
Location	
C/A/T/D/S/R	1/2/2/1/10/7
Size, mm, median [range]	40 [17–80]
Differentiation	
Well‐differentiated	2 (8.7)
Moderately differentiated	21 (91.3)
Depth of invasion	
Muscularis propria/subserosa	5/18
Positive for venous invasion	13 (56.5)
Positive for lymphatic invasion	7 (30.4)
Positive for lymph node metastasis	13 (56.5)
TNM stage	
I/II/III/IV	4/6/13/0

*Note*: C, cecum; A, ascending colon; T, transverse colon; D, descending colon; S, sigmoid colon; R, rectum. Values represent *n* (%) unless noted otherwise.

All participants in this study provided written informed consent, and the study was approved by the Ethical Committee of Iwate Medical University (HG2021‐023).

### 2.1 Crypt isolation technique

For the crypt isolation method, we first prepared both normal tissues and tumor samples containing the invasive front from resected CRC specimens.^14^ Tumor samples were obtained primarily from three components within the same tumor: HGIL, ISL, and IFL. There may be difficulty with this method in discriminating these three tumor components macroscopically before fixation. Therefore, we carefully confirmed the borders of the three components and collected each component separately. Even with careful collection, minimal contamination among the components may be inevitable. After fixation, histological analysis confirmed that cancer glands were isolated from each component without contamination of the adjacent areas. Discohesive and solid components were often excluded due to their poor isolation from cancer tissue. Normal colonic mucosa was collected from the most distal portion of the colon. The tumor and normal tissues were subjected to a crypt isolation method according to a previously reported method.^14^ Briefly, fresh mucosa and tumor specimens were minced into small pieces using a razor and incubated at 37°C for 30 min in calcium‐ and magnesium‐free Hanks' balanced salt solution (CMF) containing 30 mM EDTA. Next, the tissues were stirred in CMF for 30–40 min. The isolated glands were immediately fixed in 70% ethanol and stored at 4°C until DNA extraction.

### 2.2 DNA extraction

DNA from normal and tumor glands of each patient was extracted by standard SDS–proteinase K treatment. The extracted DNA was resuspended in TE buffer (10 mM Tris–HCl, 1 mM EDTA [pH 8.0]).

### 2.3 Analysis of microsatellite instability (MSI)

MSI status was determined based on a National Cancer Institute panel of five markers: BAT25, BAT26, D2S123, D5S346, and D17S250. MSI‐high status was defined as two or more markers being unstable and MSI‐low status as one marker being unstable; microsatellite stability was defined as the absence of instable markers.^15^

### 2.4 SNP array analysis

We examined SCNAs using the Cytoscan HD (Thermo Fisher Scientific) platform, which contains over 1.9 million non‐polymorphic markers and over 740,000 SNP markers, with mean intragenic and intergenic marker spacings of 880 and 1737 bp, respectively. These platforms are composed of microarrays containing non‐polymorphic probes for copy number variations from coding and noncoding regions of the human genome, as well as polymorphic SNP probes. All procedures were performed according to the manufacturer's instructions. Slides were analyzed using the GeneChip® Scanner 3000 7G (Thermo Fisher Scientific) and Chromosome Analysis Suite software (Thermo Fisher Scientific). We examined SCNAs using gene loci rather than chromosomal loci.

### 2.5 Classification of SCNAs

We classified the SCNAs into three subtypes: gain, loss of heterozygosity (LOH), and copy neutral LOH (CN‐LOH). LOH was defined as a gross chromosomal change resulting in loss of an entire gene and its surrounding region. A gain was defined as a gross chromosomal change resulting in gain of an entire gene and its surrounding region. CN‐LOH was defined as LOH without a copy number change (copy number = 2). The detailed classification criteria are described elsewhere.^8^, ^9^

### 2.6 Tissue microarray construction

**FIGURE 1:**
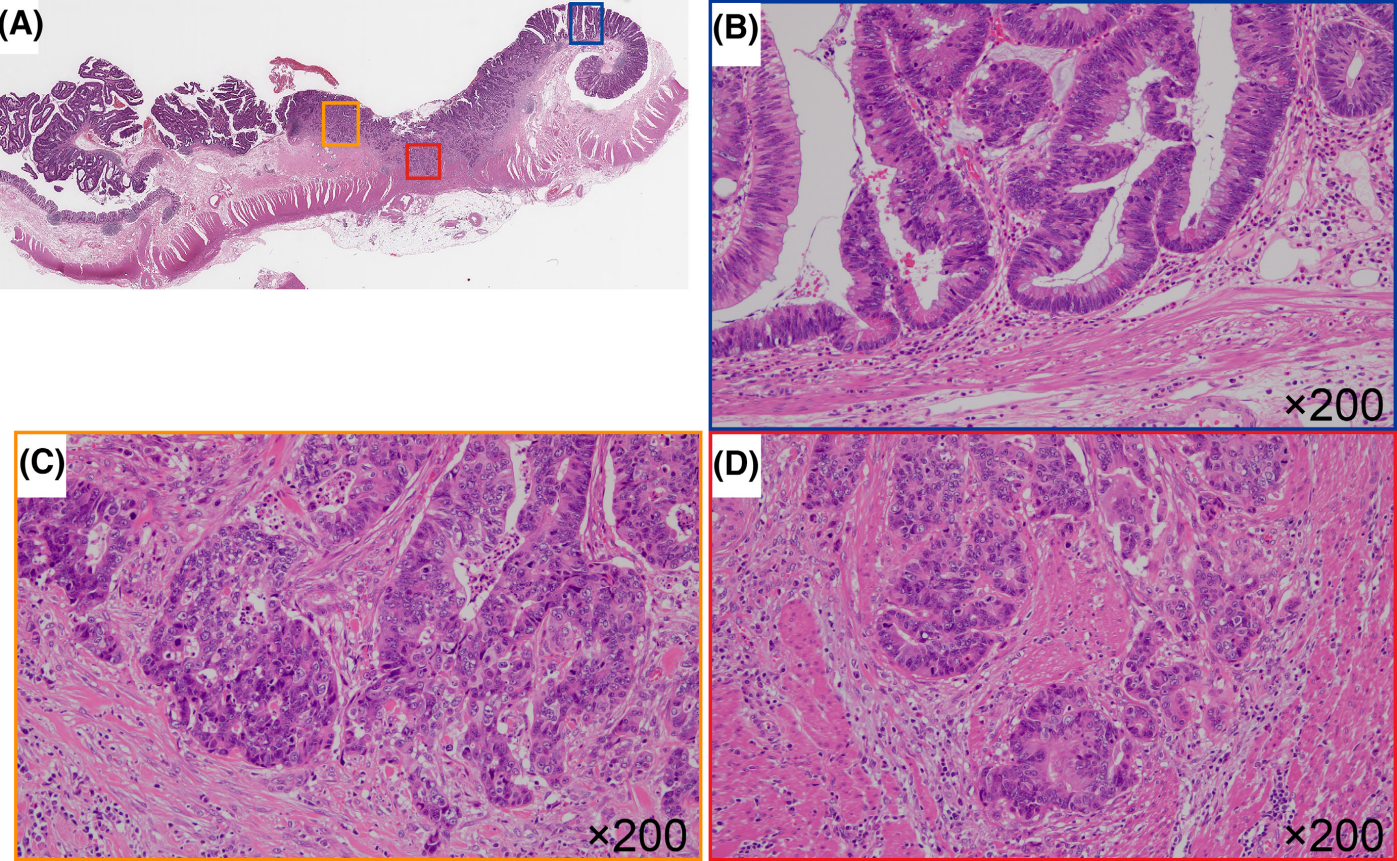
Representative histological features of colorectal cancer (CRC) including the intramucosal, invasive submucosal, and invasive front components. (A) Low‐power view of the whole tumor, (B) high‐grade intramucosal lesion, (C) invasive submucosal lesion, and (D) invasive front lesion.

Tissue microarrays were created using a manual tissue array (Azumaya Co.). The HGIL, ISL, and IFL components were obtained separately from the same tumor (Figure 1) and then subjected to immunohistochemistry. Tissue cores (3 mm) were collected from each lesion type and placed into a recipient block containing 19 cores comprising 18 cancer tissues and 1 control tissues (normal stroma surrounding the CRC). After construction, 3‐μm sections were cut and stained with hematoxylin and eosin to verify the histological diagnosis. Serial sections were cut from the microarray block for immunohistochemical staining.

### 2.7 Immunohistochemistry

Immediately after excision, tumor specimens were fixed in 10% neutral buffered formalin, embedded in paraffin wax, cut into 3‐μm‐thick sections, and stained with hematoxylin and eosin for routine pathological diagnosis. For immunohistochemical staining, additional 3‐μm‐thick sections were cut from the paraffin‐embedded tissue and placed on poly‐l‐lysine‐coated glass slides.

Immunohistochemistry was performed using the DAKO Envision+ system, consisting of dextran polymers conjugated to horseradish peroxidase (DAKO), as described previously.^16^ Hematoxylin was used as the counterstain. The antibodies used are shown in Table S1.

### 2.8 Assessment of immunohistochemical results

The immunostaining intensity and immunostained area were evaluated separately. The immunostaining intensity in cancer cells was classified as negative, weak, moderate, or strong. The immunostained area in fusiform stromal cells was semi‐quantified as 0%, 1%–25%, 26%–50%, or 51%–100%. The sum of the intensity and area scores was calculated and compared among the different tumor components.

### 2.9 Statistical analysis

Differences in clinicopathologic variables among groups were analyzed by Fisher's exact test using JMP Pro 16.1 software package for Windows (SAS). Differences in age and tumor size distribution were evaluated using the Mann–Whitney *U* test in JMP Pro 16.1. Comparisons among multiple subgroups were performed using the Friedman test. If significant differences among multiple groups were found, differences between two groups were analyzed using the Wilcoxon signed rank test with Bonferroni correction.

## 3 RESULTS

The MSI status of all samples was determined. All five markers (BAT25, BAT26, D2S123, D5S346, and D17S250) were microsatellite stable in 20 cases, and one marker (D5S346 or D2S123) was MSI in the remaining 3 cases.

The SCNAs in all chromosomes according to lesion type are shown in Figure S1. In the HGIL component, the gain regions detected in over 30% of the cases were located at 20q, 7q, 14q, 13q, 7p, 20p, 8q, and 8p, the LOH regions at 18q, 17p, and 18p, and the CN‐LOH regions at 3p, 1p, and 15q. In the ISL component, the gain regions detected in over 50% of cases were located at 20q, 8q, 7p, 20p, 7q, 13q, 8p, and 16p, and the LOH events were located at 18q, 17p, and 18p, whereas no CN‐LOH events occurred in over 50% of cases. The detailed data can be found in Table S2.

In the HGIL component, the mean number of total chromosomal aberrations per patient was 76, including 52 (range: 1–388) gains, 17 (0–136) LOH events, and 14 (2–70) CN‐LOH events. In the ISL component, the mean number of total chromosomal aberrations per patient was 283, including 151 (range: 3–594) gains, 66 (0–216) LOH events, and 30 (0–141) CN‐LOH events.

### 3.1 Differences in SCNAs at the gene level between HGIL and ISL components

We examined significant differences in the gene copy number alterations (GCNAs) among the HGIL, ISL, and IFL components. When the HGIL and ISL components were compared, the number of gains was 964, with a significant difference found between the components. There were four gene LOH and nine CN‐LOH events (Table S3). Regarding the differences in GCNA number between the ISL and IFL components, there were 1146 gene gains and 1 LOH event in both components combined (Table S3). However, CN‐LOH was not detected. The most frequent loci with gains were 8q, 16p, and 20p/q (Table S4). Detailed data are shown in Tables S4 and S5.

**FIGURE 2:**
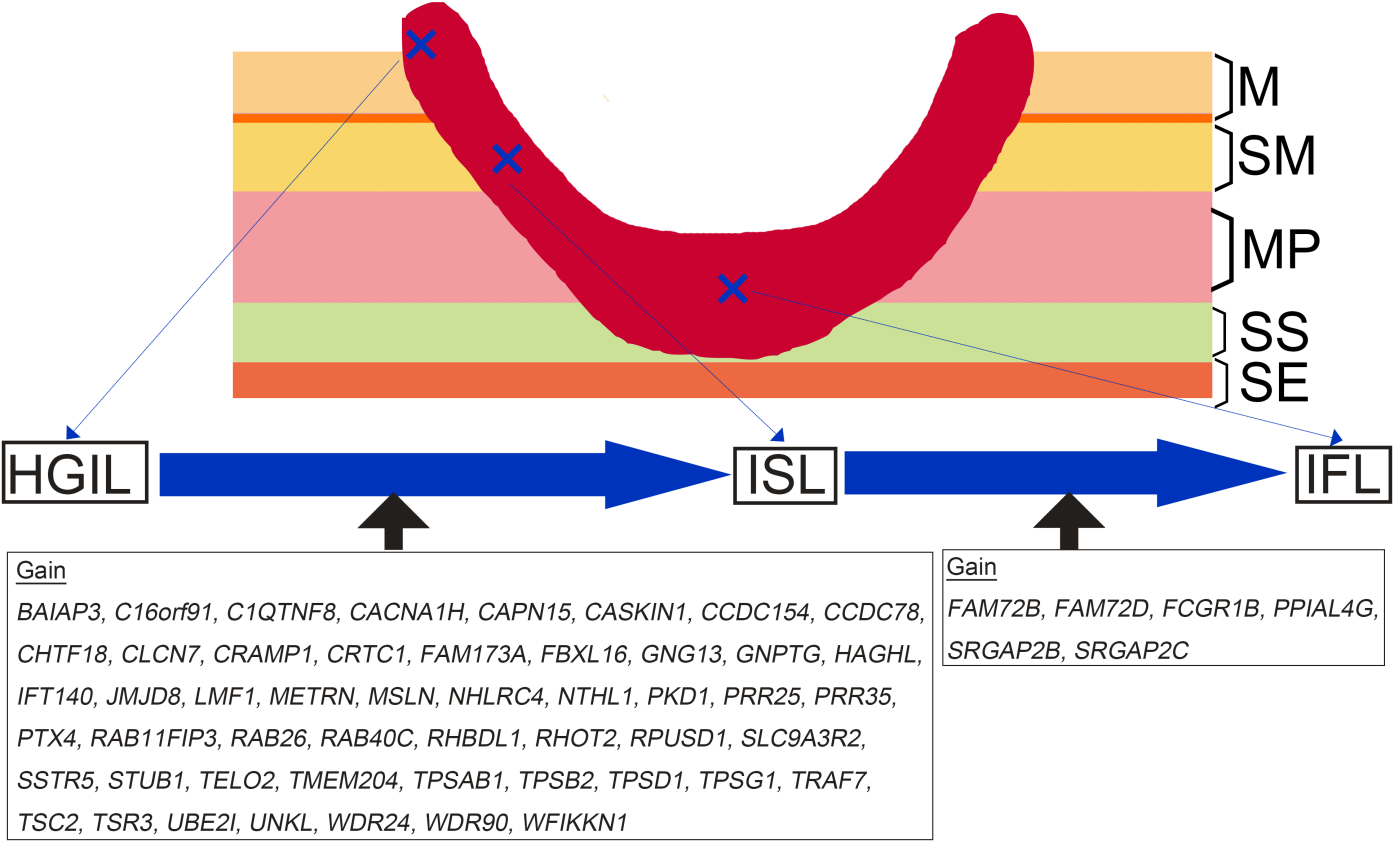
Schematic diagram of the progression from a high‐grade intramucosal lesion (HGIL) to an invasive submucosal lesion (ISL) and ultimately to an invasive front lesion (IFL). Fifty‐one amplified genes responsible for the progression from high‐grade intramucosal to invasive submucosal lesions are listed in the left column. Six amplified genes closely associated with the progression from submucosal to invasive front lesions are listed in the right column.

To identify candidate genes distinguishing the two components, we selected those genes with a dramatic as well as statistical difference in gain frequency between the two components (e.g., <5% in lesion A vs. >50% in lesion B, *p* < 0.01). Although genes with such differences in gain frequency between the HGIL and ISL components were detected (51 genes), no LOH or CN‐LOH at the gene level was found (Figure 2). Among the 51 genes closely associated with the progression from HGIL to submucosal invasive cancer, we selected the following 3 reported to have a close relationship with colorectal carcinogenesis: target of rapamycin kinase complex 1 (TORC1; 19p13.11), mesothelin (MSLN; 16p13.3), and STIP1 homology and U‐box containing protein 1 (STUB1; 16p13.3).

Finally, we compared GCNA numbers between ISL and IFL components using the same criteria. The significant genes showing a gain were *FAM72B* (family with sequence similarity 72 member B; 1p11.2), *FAM72D* (family with sequence similarity 72 member D; 1p21.1), *FCGR1B* (Fc fragment of IgG receptor Ib; 1p11.2), *PPIAL4G* (peptidylprolyl isomerase A like 4G; 1q21.2), *SRGAP2B* (SLIT‐ROBO Rho GTPase 2B 1q21.2), and *SRGAP2C* (SLIT‐ROBO Rho GTPase 2C; 1q21.2). However, no LOH or CN‐LOH at the gene level was detected between the two components.

### 3.2 Differences in number of GCNAs among components

**FIGURE 3:**
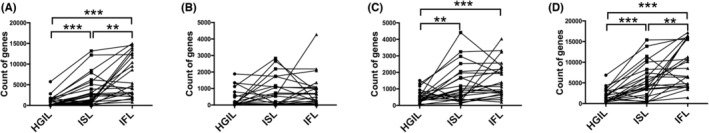
Numbers of SCNAs in the high‐grade intramucosal lesion (HGIL), invasive submucosal lesion (ISL), and invasive front lesion (IFL) components of 23 tumors according to SCNA type. (A) Gain, (B) loss of heterozygosity (LOH), (C) copy‐neutral loss of heterozygosity (CN‐LOH), and (D) total SCNAs. *p < 0.05, **p < 0.01, ***p < 0.001.

There were significant differences in the total number of GCNAs and mean number of somatic gains between HGIL and ISL components and between ISL and IFL components (*p* < 0.01). In addition, a significant difference in the mean number of gene gains between HGIL and IFL components was found. Although the LOH frequency was similar among the three subgroups, there were significant differences in the mean number of CN‐LOH events between HGIL and ISL components and between HGIL and IFL components (*p* < 0.01). These data are shown in Figure 3.

### 3.3 Immunohistochemical assessment of affected genes

**FIGURE 4:**
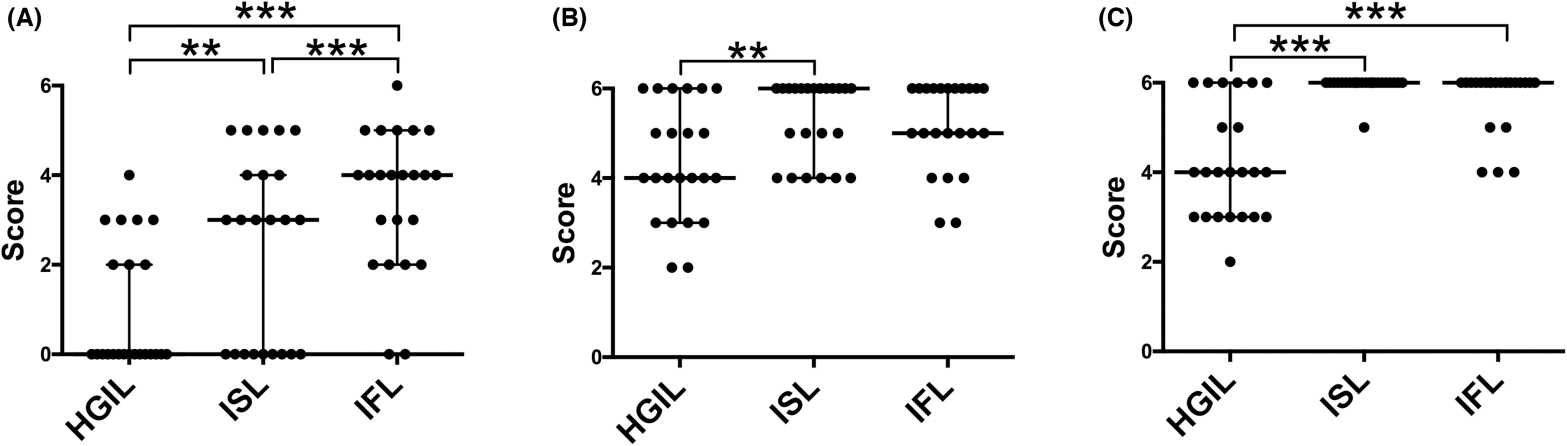
Comparison of the immunohistochemical scores of MSLN (A), TORC1 (B), and STUB1 (C) among the high grade intramucosal lesion (HGIL), invasive submucosal lesion (ISL), and invasive front lesion (IFL) components. *p < 0.05, **p < 0.01, ***p < 0.001.

**FIGURE 5:**
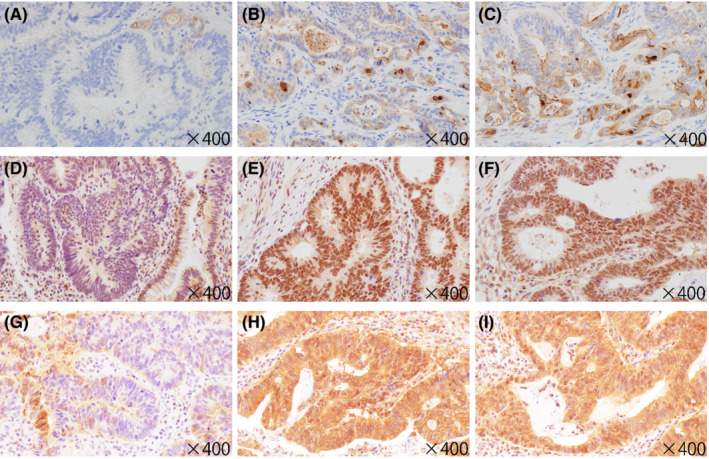
Immunohistochemical expression of MSLN, TORC1, and STUB1 in the high‐grade intramucosal lesion (HGIL), invasive submucosal lesion (ISL), and invasive front lesion (IFL) components. (A–C) Expression of MSLN in HGIL (score 0) (A), ISL (score 3) (B), and IFL (score 4) (C). (D–F) Expression of TORC1 in HGIL (score 2) (D), ISL (score 6) (E), and IFL (score 6) (F). (G–I) Expression of STUB1 in HGIL (score 3) (G), ISL (score 6) (H), and IFL (score 6) (I).

Although three candidate antibodies targeting TORC1, MSLN or STUB1 were used to examine immunohistochemical expression, we could not find antibodies targeting FAM72B, FAM72D, FCGR1B, PPIAL4G, SRGAP2B, or SRGAP2 that are suitable for immunohistochemistry. Therefore, we examined the immunohistochemical expression of only TORC1, MSLN, and STUB1. We found significant differences in the immunohistochemical scores of all three markers between the HGIL and ISL components (Figure 4). Despite significant differences in the immunohistochemical score of MSLN between the ISL and IFL components, there was no significant difference in the immunohistochemical score of TORC1 or STUB1 between these two components. Representative immunohistochemical features are shown in Figure 5.

## 4 DISCUSSION

Most previous studies focused on the effects of SCNAs in protein‐coding genes and their association with the affected genes.^17^, ^18^, ^19^ Analysis of publicly available TCGA data clearly shows that certain tumor types have genomic regions affected by SCNAs, which may be closely associated with tumor progression.^5^ Although SCNA development has been investigated extensively in human cancers of different stages,^10^, ^12^ very little is known regarding how SCNAs accumulate during CRC progression within the same tumor and how such accumulation affects cancer invasion.^8^ In this study, tracking SCNA development from HGIL to IFL, via ISL as the intermediate stage, will help elucidate the mechanism of cancer progression.

Recent studies have shown that advanced adenomas including HGIL have a high frequency of SCNAs,^8^ whereas the number of SCNAs might be higher in metastatic than primary CRC.^20^, ^21^ Thus, progression from an early‐stage to invasive lesion in CRC seems to involve stepwise accumulation of SCNAs. SCNAs, primarily classified into gain and loss types, may result from different genomic events. Oncogenes are often found in amplified genomic regions, whereas tumor suppressors tend to be located within deleted genomic segments. According to previous studies, high‐level amplification or homozygous deletion may be necessary to activate an oncogene or inactivate a tumor suppressor gene. Associated amplifications might indicate that the affected genes function synergistically to promote oncogenesis. In our study, GCNA gains and total GCNAs had accumulated within the same tumor, suggesting that accumulation of overall GCNAs and gene gains plays a major role in cancer progression.

Previous studies showed that many loci associated with gains, including 1p/q, 3p, 4p, 5p, 7p, 9p/q, 11p, 12p/q, 2p, 10q, 13q and 15q, promote cancer progression from IMC to an early‐stage invasive lesion in separate lesions (adenoma, IMC, and early invasive CRC).^10^, ^12^, ^17^, ^18^ However, SCNAs identified as contributing to invasion beyond the mucosa in separate lesions differ from those identified in the same lesion. In this study, SCNAs responsible for cancer progression present within the same tumor occurred at 8q, 16p and 20p/q. Such discrepancy is important for evaluating colorectal carcinogenesis. We found that genes located at 16p13.3 play an important role in neoplastic progression from HGIL to ISL and may aid elucidation of early colorectal carcinogenesis.

The frequency of a specific genetic aberration in a tumor sample may be correlated with recurrence‐free survival or overall survival, possibly leading to that aberration's use as a biomarker for therapeutic decisions.^22^, ^23^ However, no adequate markers exist to predict disease recurrence in colorectal cancer. A previous study showed that gain of the 16p13.3 locus was linked to worse recurrence‐free survival in their patient cohort.^24^ In addition, loss of RBFOX1 (*RNA Binding Fox‐1 Homolog 1*, 16p13.3) was found only in patients without recurrence.^24^ The current results support this finding and help elucidate molecular mechanisms underlying tumor progression.

It is important for pathologists and gastroenterologists to evaluate the molecular mechanism underlying progression from HGIL to ISL.^8^ Although many studies have addressed this mechanism, an effective marker has not been identified. The current model proposing that the process of CRC progression can be seen within the same tumor is novel for evaluating the essential molecular alterations involved in tumor progression. Herein, we identified GCNAs that play an important role in the progression from HGIL to ISL or from ISL to IFL. Among affected genes, we searched for those potentially contributing to colorectal carcinogenesis. Three genes with gains (*TORC1*, *MSLN*, and *STUB1*) were selected as potential markers of the progression of a CRC tumor from HGIL to ISL. However, these candidate genes were not found among the genes identified as promoting progression from ISL to IFL.

STUB1, which is a co‐chaperone protein and U‐box‐containing E3 ligase, is involved in the degradation of several oncogenic proteins, including ErbB2, hypoxia inducible factor 1α, and c‐Myc, and consequently acts as a tumor suppressor.^25^, ^26^, ^27^ Consequently, STUB1 is downregulated in human CRC, breast cancer, and gastric cancer.^25^ In this study, however, *STUB1* was amplified at the gene level according to a SNP array and was overexpressed in CRC. In addition, its expression was higher in the ISL than HGIL component of the same tumor. Although the reason for this discrepancy is unclear, it may be due to differences between the in vivo and vitro experiments. Further studies are needed to resolve this discrepancy.

A recent study showed strong evidence that TORC1 activity is enhanced in mouse and human colon tumor specimens.^28^ That study also demonstrated that the transcriptional program regulated by TORC1/CREB and TORC1/AP1 complexes is activated in colonic cancer epithelial cells, and TORC1 was identified as a novel mediator of PGE2 signaling that promotes colorectal carcinogenesis and activation of the downstream pro‐tumorigenic targets in colon cancer.^28^, ^29^, ^30^ Thus, TORC1 activation might be an important molecular mechanism mediating the effects of PGE2 on colon tumor growth, inducing pro‐tumorigenic transcription of genes including *NR4A2*, *COX2*, *AREG*, and *IL‐6*. In this study, TORC1 was overexpressed in the progression from HGIL to ISL components within the same tumor, suggesting that TORC1 overexpression is a novel marker of early colorectal carcinogenesis.

MSLN is a glycophosphatidylinositol‐linked cell surface protein typically expressed in mesothelial cells. MSLN is overexpressed in several types of malignant tumors, including malignant pleural mesothelioma, ovarian cancer, pancreatic adenocarcinoma, and gastric cancer.^31^ Recently, significant overexpression of MSLN was detected in approximately 60% of CRC cases.^31^, ^32^ High expression of MSLN was associated with poor prognosis of patients with breast and lung adenocarcinomas in previous studies. Poor prognosis of CRC with a MSLN‐positive phenotype has also been reported.^32^, ^33^ According to that study, MSLN‐positive CRC patients with metastatic lesions that worsen prognosis might be good candidates for MSLN‐targeting therapeutics. Here, we found that overexpression of MSLN plays an important role in the progression from HGIL to ISL within the same tumor. This result was supported by the finding that SCNAs accumulate during progression from HGIL to ISL.

Overall, TORC1 and MSLN may be candidate markers predicting the progression from HGIL to ISL^28^, ^31^, ^32^ and STUB1 was reported to act as a tumor suppressor.^25^

There are some limitations to this study. First, the sample size was small. However, the current model evaluating CRC progression is novel. In particular, identification of the molecular mechanism underlying progression from HGIL to ISL within the same tumor is very interesting. Actually, the current model that we examined is rarely encountered in routine pathology practice. Validation will be performed in the near future. Second, this is a retrospective study. To confirm the results, a prospective study may be needed. However, although a prospective study using this model of CRC progression within the same tumor may be difficult, the markers that we examined should be confirmed in a larger number of cases. Finally, although the immunohistochemical scores of TORC1 and MSLN were significantly different among the three lesions, they showed overlap. The utility of these markers may be limited in routine practice.

In conclusion, we examined SCNAs on the gene level to help elucidate molecular mechanisms of progression from HGIL to an invasive lesion within the same tumor. We demonstrated that progression from HGIL to an invasive lesion was associated with a novel molecular profile for the microsatellite stable pathway (or chromosomal instability pathway). Our SNP array data showed that although the frequency of GCNAs detected in HGIL was low, that in IFL varied. Molecular profiling of SCNAs within the same tumor could provide novel insights into early colorectal carcinogenesis. In addition, high expression of TORC1and MSLN may predict the progression from HGIL to ISL. The relationships among SCNA events may help identify oncogenic network modules.

## AUTHOR CONTRIBUTIONS

S Yamada, the first author, constructed the figures and tables and performed the statistical analyses. M Osakabe assisted with the statistical analyses. N Uesugi and N Yanagawa helped with the pathological examination. T Matsumoto provided clinical support during preparation of the manuscript. H Suzuki supported the molecular examination. T Sugai, the corresponding author, contributed to the preparation of the manuscript, including all aspects of the data collection and analysis.

## FUNDING INFORMATION

No funding was received for this study.

## CONFLICT OF INTEREST

We declare that we have no conflicts of interest.

## ETHICAL APPROVAL AND CONSENT TO PARTICIPATE

Informed consent was obtained from each patient according to institutional guidelines, and the research protocols were approved by the ethics committee of Iwate Medical University Hospital (approval number HG2021‐023).

## HUMAN RIGHTS STATEMENT AND INFORMED CONSENT

All procedures were performed in accordance with the ethical standards of the Iwate Medical University and with the Declaration of Helsinki. Informed consent (approval by the institutional review board of Iwate Medical University) was obtained from all patients included in the study.

## CONSENT FOR PUBLICATION

Not applicable.

## Supporting information


Table S1
Table S2
Table S3
Table S4
Table S5

Figure S1

## Data Availability

The datasets used and/or analyzed in the current study are available from the corresponding author upon reasonable request.

## References

[1] Vogelstein B , Fearon ER , Hamilton SR , et al. Genetic alterations during colorectal tumor development. N Engl J Med. 1988;319:525‐532. 2841597 10.1056/NEJM198809013190901

[2] Nagtegaal ID , Arends M , Salto‐Tellez M . Colorectal Adenocarcinoma. WHO Classification of Tumours of the Digestive System. International Agency for Research on Cancer; 2019:177‐182.

[3] Pino MS , Chung DC . The chromosomal instability pathway in colon cancer. Gastroenterology. 2010;138:2059‐2072. doi:10.1053/j.gastro.2009.12.065 20420946 PMC4243705

[4] Jass JR , Whitehall VL , Young J , Leggett BA . Emerging concepts in colorectal neoplasia. Gastroenterology. 2002;123:862‐876. doi:10.1053/gast.2002.35392 12198712

[5] Cancer Genome Atlas Network . Comprehensive molecular characterization of human colon and rectal cancer. Nature. 2012;487:330‐337. 22810696 10.1038/nature11252 PMC3401966

[6] Guinney J , Dienstmann R , Wang X , et al. The consensus molecular subtypes of colorectal cancer. Nat Med. 2015;21:1350‐1356. doi:10.1038/nm.3967 26457759 PMC4636487

[7] Ogino S , Goel A . Molecular classification and correlates in colorectal cancer. J Mol Diagn. 2008;10:13‐27. doi:10.2353/jmoldx.2008.070082 18165277 PMC2175539

[8] Sugai T , Osakabe M , Habano W , et al. A genome‐wide analysis of the molecular alterations occurring in the adenomatous and carcinomatous components of the same tumor based on the adenoma‐carcinoma sequence. Pathol Int. 2021;71:582‐593. doi:10.1111/pin.13129 34263942 PMC8518074

[9] Sugai T , Osakabe M , Sugimoto R , et al. A genome‐wide study of the relationship between chromosomal abnormalities and gene expression in colorectal tumors. Genes Chromosomes Cancer. 2021;60:250‐262. doi:10.1002/gcc.22924 33258187 PMC7898915

[10] Sugai T , Eizuka M , Habano W , et al. Comprehensive molecular analysis based on somatic copy number alterations in intramucosal colorectal neoplasias and early invasive colorectal cancers. Oncotarget. 2018;9:22895‐22906. doi:10.18632/oncotarget.25112 29796160 PMC5955401

[11] Sugai T , Takahashi Y , Eizuka M , et al. Molecular profiling and genome‐wide analysis based on somatic copy number alterations in advanced colorectal cancers. Mol Carcinog. 2018;57:451‐461. doi:10.1002/mc.22769 29230882 PMC5814737

[12] Eizuka M , Sugai T , Habano W , et al. Molecular alterations in colorectal adenomas and intramucosal adenocarcinomas defined by high‐density single‐nucleotide polymorphism arrays. J Gastroenterol. 2017;52:1158‐1168. doi:10.1007/s00535-017-1317-2 28197804 PMC5666076

[13] Japanese Society for Cancer of the Colon and Rectum . Japanese Classification of Colorectal Carcinoma, Second English Edition. Kanehara Co.; 2009:30‐63.

[14] Habano W , Sugai T , Nakamura S , Yoshida T . A novel method for gene analysis of colorectal carcinomas using a crypt isolation technique. Lab Invest. 1996;74:933‐940. 8642788

[15] Boland CR , Thibodeau SN , Hamilton SR , et al. A National Cancer Institute workshop on microsatellite instability for cancer detection and familial predisposition: development of international criteria for the determination of microsatellite instability in colorectal cancer. Cancer Res. 1998;58:5248‐5257. 9823339

[16] Sugai T , Yamada N , Osakabe M , et al. Microenvironmental markers are correlated with lymph node metastasis in invasive submucosal colorectal cancer. Histopathology. 2021;79:584‐598. doi:10.1111/his.14388 33884652 PMC8518933

[17] Wang H , Liang L , Fang JY , Xu J . Somatic gene copy number alterations in colorectal cancer: new quest for cancer drivers and biomarkers. Oncogene. 2016;35:2011‐2019. doi:10.1038/onc.2015.304 26257062

[18] Xie T , D' Ario G , Lamb JR , et al. A comprehensive characterization of genome‐wide copy number aberrations in colorectal cancer reveals novel oncogenes and patterns of alterations. PLoS One. 2012;7:e42001. doi:10.1371/journal.pone.0042001 22860045 PMC3409212

[19] Lassmann S , Weis R , Makowiec F , et al. Array CGH identifies distinct DNA copy number profiles of oncogenes and tumor suppressor genes in chromosomal‐ and microsatellite‐unstable sporadic colorectal carcinomas. J Mol Med (Berl). 2007;85:293‐304. doi:10.1007/s00109-006-0126-5 17143621

[20] Hieronymus H , Murali R , Tin A , et al. Tumor copy number alteration burden is a pan‐cancer prognostic factor associated with recurrence and death. Elife. 2018;7:e37294. doi:10.7554/eLife.37294 30178746 PMC6145837

[21] Smeets D , Miller IS , O'Connor DP , et al. Copy number load predicts outcome of metastatic colorectal cancer patients receiving bevacizumab combination therapy. Nat Commun. 2018;9:4112. doi:10.1038/s41467-018-06567-6 30291241 PMC6173768

[22] Carvalho B , Postma C , Mongera S , et al. Multiple putative oncogenes at the chromosome 20q amplicon contribute to colorectal adenoma to carcinoma progression. Gut. 2009;58:79‐89. doi:10.1136/gut.2007.143065 18829976

[23] Andersen CL , Lamy P , Thorsen K , et al. Frequent genomic loss at chr16p13.2 is associated with poor prognosis in colorectal cancer. Int J Cancer. 2011;129:1848‐1858. doi:10.1002/ijc.25841 21154748

[24] Mampaey E , Fieuw A , Van Laethem T , et al. Focus on 16p13.3 locus in colon cancer. PLoS One. 2015;10:e0131421. doi:10.1371/journal.pone.0131421 26222184 PMC4519182

[25] Ballinger CA , Connell P , Wu Y , et al. Identification of CHIP, a novel tetratricopeptide repeat‐containing protein that interacts with heat shock proteins and negatively regulates chaperone functions. Mol Cell Biol. 1999;19:4535‐4545. doi:10.1128/MCB.19.6.4535 10330192 PMC104411

[26] Meacham GC , Patterson C , Zhang W , Younger JM , Cyr DM . The Hsc70 co‐chaperone CHIP targets immature CFTR for proteasomal degradation. Nat Cell Biol. 2001;3:100‐105. doi:10.1038/35050509 11146634

[27] Schumacher Y , Aparicio T , Ourabah S , et al. Dysregulated CRTC1 activity is a novel component of PGE2 signaling that contributes to colon cancer growth. Oncogene. 2016;19(35):2602‐2614. doi:10.1038/onc.2015.283 26300003

[28] Canettieri G , Coni S , Della Guardia M , et al. The coactivator CRTC1 promotes cell proliferation and transformation via AP‐1. Proc Natl Acad Sci U S A. 2009;106:1445‐1450. doi:10.1073/pnas.0808749106 19164581 PMC2635810

[29] Chen Z , Chen J , Gu Y , et al. Aberrantly activated AREG‐EGFR signaling is required for the growth and survival of CRTC1‐MAML2 fusion‐positive mucoepidermoid carcinoma cells. Oncogene. 2013;33:3869‐3877. doi:10.1038/onc.2013.348 23975434

[30] Inoue S , Tsunoda T , Riku M , et al. Diffuse mesothelin expression leads to worse prognosis through enhanced cellular proliferation in colorectal cancer. Oncol Lett. 2020;19:1741‐1750. doi:10.3892/ol.2020.11290 32194667 PMC7039175

[31] Inaguma S , Wang Z , Lasota J , et al. Comprehensive immunohistochemical study of mesothelin (MSLN) using different monoclonal antibodies 5B2 and MN‐1 in 1562 tumors with evaluation of its prognostic value in malignant pleural mesothelioma. Oncotarget. 2017;8:26744‐26754. doi:10.18632/oncotarget.15814 28460459 PMC5432294

[32] Kawamata F , Homma S , Kamachi H , et al. C‐ERC/mesothelin provokes lymphatic invasion of colorectal adenocarcinoma. J Gastroenterol. 2014;49:81‐92. doi:10.1007/s00535-013-0773-6 23512344

[33] Inaguma S , Lasota J , Felisiak‐Golabek A , et al. Histopathological and genotypic characterization of metastatic colorectal carcinoma with PD‐L1 (CD274)‐expression: possible roles of tumour micro environmental factors for CD274 expression. J Pathol Clin Res. 2017;3:268‐278. doi:10.1002/cjp2.81 29085667 PMC5653930

