# Supplementary material for: Genome‐wide analysis of colorectal cancer based on gene‐based somatic copy number alterations during neoplastic progression within the same tumor
Source: Cancer Med. 2022 Aug 3;12(4):4446–54. doi: 10.1002/cam4.5117 (PMC9972084; doi:10.1002/cam4.5117)
Supplement: Supplementary file 1 — Table S1 Table S2 Table S3 Table S4 Table S5 [file CAM4-12-4446-s002.docx]

Supplementary Table 1. Antibodies used in the present study.

| Antibody | Source | Clone | Dilution | Treatment |
| --- | --- | --- | --- | --- |
| Mesothelin | Leica | 5B2 | Ready to use | Heat retrieval (pH 6.0) |
| TORC1 | Abcam | EPR3381(2) | 1:50 | Heat retrieval (pH 6.0) |
| STUB1/CHIP | Abcam | EPR4447 | 1:400 | Heat retrieval (pH 6.0) |

Supplementary Table 2. Frequent SCNA regions in high-grade intramucosal lesions, invasive submucosal lesions and invasive front lesions of CRC tumors.

| Chromosomal region | High-grade intramucosal lesion (%) | |
| --- | --- | --- |
| **Gain** |  |  |
| 20q11.21-q13.33 | 9-12 | (39.1–52.2) |
| 7q11.21-q36.3 | 7-12 | (30.4-52.2) |
| 14q32.33 | 11 | (47.8) |
| 13q12.11-q34 | 7-9 | (30.4-39.1) |
| 7p22.3-p11.2 | 8 | (34.8) |
| 20p12.1-p11.21 | 8 | (34.8) |
| 8q12.3-q24.22 | 7-8 | (30.4-34.8) |
| 8p11.23-p11.21, 8q24.3 | 7 | (30.4) |
| **LOH** |  |  |
| 18q11.2-q23 | 8 | (34.8) |
| 17p13.3-p11.2, 18p11.32-p11.21 | 7 | (30.4) |
| **CN-LOH** |  |  |
| 3p21.31-p21.2 | 7-9 | (30.4-39.1) |
| 1p32.3 | 8 | (34.8) |
| 15q15.1-q15.3 | 7-8 | (30.4-34.8) |
| Chromosomal region | Invasive submucosal lesion (%) | |
| **Gain** |  |  |
| 20q11.21-q13.33 | 20-21 | (87.0-91.3) |
| 8q11.21-q24.3 | 13-17 | (56.5-73.9) |
| 7p22.3-p11.2, 20p13-p11.21 | 15-16 | (65.2-69.6) |
| 7q11.21-q36.3 | 13-16 | (56.5-69.6) |
| 13q12.11-q34 | 13-15 | (56.5-65.2) |
| 8p11.21, 16p13.3, 16p13.13, 16p11.2 | 12 | (52.2) |
| **LOH** |  |  |
| 18q11.2-q23 | 12-15 | (52.2-65.2) |
| 17p13.3-p11.2, 18p11.31-p11.21 | 12 | (52.2) |
| **CN-LOH** |  |  |
| None |  |  |
| Chromosomal region | Invasive front lesion(%) | |
| **Gain** |  |  |
| 7p22.3-p11.2 | 22-23 | (95.7-100.0) |
| 7q11.21-q36.3 | 20-23 | (87.0-100.0) |
| 20q11.21-q13.33 | 22 | (95.7) |
| 13q12.11-q34 | 19-22 | (82.6-95.7) |
| 8q11.21-q24.3 | 19-21 | (82.6-91.3) |
| 20p13-p11.21 | 15-18 | (65.2-78.3) |
| 16p13.3-p11.2 | 13-17 | (56.5-73.9) |
| 2p25.3-p11.2 | 13-16 | (56.5-69.6) |
| 2q11.2-q37.3 | 12-16 | (52.2-69.6) |
| 14q32.33 | 15 | (65.2) |
| 1q21.3-q44, 8p11.23-p11.21 | 14-15 | (60.9-65.2) |
| 19q12-q13.43 | 13-15 | (56.5-65.2) |
| 16q12.1-q22.3 | 12-14 | (52.2-60.9) |
| 3q21.1-q29 | 13 | (56.5) |
| 5p15.33-5p12, 6p25.3-p24.2, 12p13.33-p13.31, 12q12-q24.33, 19p13.3-p12 | 12-13 | (52.2-56.5) |
| 9p24.3-p13.2, 10p12.31, 10p11.21, 17q21.33, 17q23.1-q24.3, 17q25.3 | 12 | (52.2) |
| **LOH** |  |  |
| 18q12.1-q23 | 14-15 | (60.9-65.2) |
| 17p13.3-p11.2 | 13-14 | (56.5-60.9) |
| 8p23.3-p21.2 | 12-13 | (52.2-56.5) |
| 18p11.31-p11.21 | 12 | (52.2) |
| **CN-LOH** |  |  |
| None |  |  |

SCNA, somatic copy number alteration; LOH, loss of heterozygosity; CN-LOH, copy neutral loss of heterozygosity.

For example, 9-12 (39.1-52.2) means “9 divided 23 is 39.1%” and 12 divided 23 is 5 2.2%, respectively.

Supplementary Table 3. The number of somatic copy number alterations with significant differences between the high-grade intramucosal lesions and invasive submucosal lesions and between the invasive submucosal lesions and invasive front lesions.

|  | HGIL vs. ISL | ISL vs. IFL |
| --- | --- | --- |
| Gain | 964 | 1146 |
| LOH | 4 | 1 |
| CN-LOH | 9 | 0 |

HGIL, high-grade intramucosal lesion; ISL, invasive submucosal lesion; IFL, invasive front lesion; LOH, loss of heterozygosity; CN-LOH, copy neutral loss of heterozygosity.

Supplementary Table 4. Somatic copy number alterations with significant differences between the high-grade intramucosal lesions and invasive submucosal lesions (964 genes with gains, 4 genes with LOH; 9 genes with CN-LOH)

| Genes (locus) | HGIL positive (%) | | ISL positive (%) | | *p* - value |
| --- | --- | --- | --- | --- | --- |
| **Gain** |  |  |  |  |  |
| CAPN15 (16p13.3), CCDC78 (16p13.3), CHTF18 (16p13.3), FAM173A (16p13.3), FBXL16 (16p13.3), GNG13 (16p13.3), HAGHL (16p13.3), JMJD8 (16p13.3), LMF1 (16p13.3), METRN (16p13.3), MSLN (16p13.3), NHLRC4 (16p13.3), PRR25 (16p13.3), PRR35 (16p13.3), RAB11FIP3 (16p13.3), RAB40C (16p13.3), RHBDL1 (16p13.3), RHOT2 (16p13.3), RPUSD1 (16p13.3), STUB1 (16p13.3), WDR24 (16p13.3), WDR90 (16p13.3), WFIKKN1 (16p13.3) | 0 | (0.0) | 13 | (56.5) | 0.0026 |
| BAIAP3 (16p13.3), C16orf91 (16p13.3), C1QTNF8 (16p13.3), CACNA1H (16p13.3), CCDC154 (16p13.3), GNPTG (16p13.3), SSTR5 (16p13.3), TPSAB1 (16p13.3), TPSB2 (16p13.3), TPSD1 (16p13.3), TPSG1 (16p13.3), TSR3 (16p13.3), UBE2I (16p13.3), UNKL (16p13.3) | 0 | (0.0) | 12 | (52.2) | 0.0045 |
| CLCN7 (16p13), CASKIN1 (16p13.3), CRAMP1 (16p13.3), IFT140 (16p13.3), NTHL1 (16p13.3), PKD1 (16p13.3), PTX4 (16p13.3), RAB26 (16p13.3), SLC9A3R2 (16p13.3), TELO2 (16p13.3), TMEM204 (16p13.3), TRAF7 (16p13.3), TSC2 (16p13.3), CRTC1 (19p13.11) | 1 | (4.3) | 12 | (52.2) | 0.0077-0.0167 |
| MIR1-1HG (20q13.33), SLCO4A1-AS1 (20q13.33) | 7 | (30.4) | 21 | (91.3) | 0.0015 |
| PLEC (8q24), CCDC166 (8q24.3), EEF1D (8q24.3), EPPK1 (8q24.3), EXOSC4 (8q24.3), FAM83H (8q24.3), GPAA1 (8q24.3), GRINA (8q24.3), GSDMD (8q24.3), MROH6 (8q24.3), NAPRT (8q24.3), NRBP2 (8q24.3), OPLAH (8q24.3), PARP10 (8q24.3), PUF60 (8q24.3), SCRIB (8q24.3), SPATC1 (8q24.3), TIGD5 (8q24.3), TSTA3 (8q24.3), ZNF623 (8q24.3), ZNF707 (8q24.3) | 4 | (17.4) | 17 | (73.9) | 0.0026 |
| RPS21 (20q13.3), CABLES2 (20q13.33), GATA5 (20q13.33), RBBP8NL (20q13.33), SLCO4A1 (20q13.33) | 8 | (34.8) | 21 | (91.3) | 0.0026 |
| FERMT1 (20p12.3), ADAM33 (20p13), ATRN (20p13), C20orf27 (20p13), CDC25B (20p13), CENPB (20p13), HSPA12B (20p13), LOC388780 (20p13), SIGLEC1 (20p13), SIRPB1 (20p13), SIRPD (20p13), SPEF1 (20p13), GFRA4 (20p13-p12), TGM3 (20q11.2) | 2 | (8.7) | 14 | (60.9) | 0.0045 |
| ZNF16 (8q24), ZNF7 (8q24), ST3GAL1 (8q24.22), ARHGAP39 (8q24.3), COMMD5 (8q24.3), CYC1 (8q24.3), MAF1 (8q24.3), NDRG1 (8q24.3), RPL8 (8q24.3), SHARPIN (8q24.3), WDR97 (8q24.3), ZNF250 (8q24.3), ZNF251 (8q24.3), ZNF34 (8q24.3), ZNF517 (8q24.3) | 4 | (17.4) | 16 | (69.6) | 0.0045 |
| BCL2L1 (20q11.21) | 7 | (30.4) | 19 | (82.6) | 0.0045 |
| NCOA3 (20q12) | 8 | (34.8) | 20 | (87.0) | 0.0045 |
| STX16-NPEPL1 (20q), NTSR1 (20q13), LAMA5 (20q13.2-q13.3), BIRC7 (20q13.3), COL9A3 (20q13.3), EEF1A2 (20q13.3), KCNQ2 (20q13.3), OGFR (20q13.3), PTK6 (20q13.3), STMN3 (20q13.3), NPEPL1 (20q13.32), STX16 (20q13.32), ADRM1 (20q13.33), ARFGAP1 (20q13.33), BHLHE23 (20q13.33), CHRNA4 (20q13.33), COL20A1 (20q13.33), DIDO1 (20q13.33), DPH3P1 (20q13.33), GID8 (20q13.33), GMEB2 (20q13.33), HELZ2 (20q13.33), HRH3 (20q13.33), MRGBP (20q13.33), NKAIN4 (20q13.33), OSBPL2 (20q13.33), PPDPF (20q13.33), SLC17A9 (20q13.33), TCFL5 (20q13.33), YTHDF1 (20q13.33) | 9 | (39.1) | 21 | (91.3) | 0.0045 |
| ELFN1 (7p22.3), INTS1 (7p22.3), MAFK (7p22.3), MICALL2 (7p22.3), PSMG3 (7p22.3), TMEM184A (7p22.3), ADGRB1 (8q24.3), MAFA (8q24.3), RHPN1 (8q24.3), TSNARE1 (8q24.3), ZC3H3 (8q24.3) | 5 | (21.7) | 17 | (73.9) | 0.0045-0.0098 |
| SOX8 (16p13.3) | 0 | (0.0) | 11 | (47.8) | 0.0077 |
| ITPA (20p), SLC4A11 (20p12), AP5S1 (20p13), C20orf194 (20p13), C20orf202 (20p13), NSFL1C (20p13), PSMF1 (20p13), SIRPB2 (20p13), TMEM74B (20p13) | 2 | (8.7) | 13 | (56.5) | 0.0077 |
| LRRN4 (20p12.3), C20orf96 (20p13), DEFB128 (20p13), DEFB129 (20p13), DEFB132 (20p13), EBF4 (20p13), IDH3B (20p13), NOP56 (20p13), PDYN (20p13), SIRPA (20p13), SIRPG (20p13), SNRPB (20p13), TGM6 (20p13), TMC2 (20p13), ZNF343 (20p13) | 3 | (13.0) | 14 | (60.9) | 0.0077 |
| KCNS2 (8q22), MATN2 (8q22), RPL30 (8q22), LAPTM4B (8q22.1), MTDH (8q22.1), POP1 (8q22.1), ERICH5 (8q22.2), NIPAL2 (8q22.2), STK3 (8q22.2), BAALC (8q22.3), CTHRC1 (8q22.3), DCAF13 (8q22.3), RIMS2 (8q22.3), SLC25A32 (8q22.3), FZD6 (8q22.3-q23.1), CPSF1 (8q24.23), ADCK5 (8q24.3), BOP1 (8q24.3), C8orf33 (8q24.3), CYHR1 (8q24.3), DGAT1 (8q24.3), FBXL6 (8q24.3), FOXH1 (8q24.3), HGH1 (8q24.3), HSF1 (8q24.3), KIFC2 (8q24.3), MROH1 (8q24.3), PPP1R16A (8q24.3), SCRT1 (8q24.3), SCX (8q24.3), SLC39A4 (8q24.3), SLC52A2 (8q24.3), TMEM249 (8q24.3), TONSL (8q24.3), VPS28 (8q24.3) | 4 | (17.4) | 15 | (65.2) | 0.0077 |
| SSTR4 (20p11.2), THBD (20p11.2), CD93 (20p11.21), CSTL1 (20p11.21), GZF1 (20p11.21), LINC00656 (20p11.21), NAPB (20p12.3-p11.21), NXT1 (20p12-p11.2) | 6 | (26.1) | 17 | (73.9) | 0.0077 |
| ID1 (20q11), POFUT1 (20q11), COX4I2 (20q11.21), DEFB116 (20q11.21), KIF3B (20q11.21), PLAGL2 (20q11.21), NNAT (20q11.2-q12) | 7 | (30.4) | 18 | (78.3) | 0.0077 |
| AAR2 (20pter-q12), ERGIC3 (20pter-q12), SCAND1 (20q11.1-q11.23), GDF5 (20q11.2), TPX2 (20q11.2), DUSP15 (20q11.21), FOXS1 (20q11.21), NOL4L (20q11.21), RBM12 (20q11.21), SPAG4 (20q11.21), C20orf173 (20q11.22), CEP250 (20q11.22), NFS1 (20q11.22), RBM39 (20q11.22), ROMO1 (20q11.22), PHF20 (20q11.22-q11.23), BPI (20q11.23), CNBD2 (20q11.23), DLGAP4 (20q11.23), KIAA1755 (20q11.23), LBP (20q11.23), RPRD1B (20q11.23), TTI1 (20q11.23), VSTM2L (20q11.23), EPB41L1 (20q11.2-q12), GDAP1L1 (20q12), TGM2 (20q12), ELMO2 (20q13), CDH22 (20q13.1), SLC2A10 (20q13.1), OSER1 (20q13.11), JPH2 (20q13.12), L3MBTL1 (20q13.12), OCSTAMP (20q13.12), SLC13A3 (20q13.12), SLC35C2 (20q13.12), SULF2 (20q13.12), TOX2 (20q13.12), ZNF334 (20q13.12), MYLK2 (20q13.31) | 8 | (34.8) | 19 | (82.6) | 0.0077 |
| TPD52L2 (20q13.2-q13.3), ARFRP1 (20q13.3), RTEL1 (20q13.3), SS18L1 (20q13.3), ZGPAT (20q13.3), ABHD16B (20q13.33), LSM14B (20q13.33), MTG2 (20q13.33), PSMA7 (20q13.33), SLC2A4RG (20q13.33), ZBTB46 (20q13.33) | 9 | (39.1) | 20 | (87.0) | 0.0077 |
| NPBWR2 (20q13.3), LKAAEAR1 (20q13.33), MYT1 (20q13.33), OPRL1 (20q13.33), PRPF6 (20q13.33), RGS19 (20q13.33), SOX18 (20q13.33), TCEA2 (20q13.33) | 10 | (43.5) | 21 | (91.3) | 0.0077 |
| UNCX (7p22.3), CYP11B1 (8q21), CYP11B2 (8q21-q22), LY6D (8q24), PSCA (8q24.2), WISP1 (8q24.22), ARC (8q24.3), C8orf31 (8q24.3), GLI4 (8q24.3), GML (8q24.3), GPIHBP1 (8q24.3), GPR20 (8q24.3), JRK (8q24.3), LY6E (8q24.3), LY6H (8q24.3), LY6K (8q24.3), LYNX1 (8q24.3), LYPD2 (8q24.3), MROH5 (8q24.3), PTP4A3 (8q24.3), SLURP1 (8q24.3), THEM6 (8q24.3), TOP1MT (8q24.3), ZFP41 (8q24.3), ZNF696 (8q24.3) | 5 | (21.7) | 16 | (69.6) | 0.0077-0.0167 |
| EME2 (16p13.3), IGFALS (16p13.3), MAPK8IP3 (16p13.3), MRPS34 (16p13.3), NPW (16p13.3), NUBP2 (16p13.3), SPSB3 (16p13.3), ZNF598 (16p13.3), NME3 (16q13.3) | 1 | (4.3) | 11 | (47.8) | 0.0133 |
| CCZ1B (7p22.1), AVP (20p13), DDRGK1 (20p13), FKBP1A (20p13), FKBP1A-SDCBP2 (20p13), LZTS3 (20p13), RAD21L1 (20p13), SDCBP2 (20p13), SNPH (20p13), UBOX5 (20p13) | 2 | (8.7) | 12 | (52.2) | 0.0133 |
| MTSS1 (8p22), NDUFB9 (8q13.3), TSPYL5 (8q22.1), CPQ (8q22.2), MAL2 (8q23), TMEM74 (8q23.1), SNTB1 (8q23-q24), COLEC10 (8q23-q24.1), HHLA1 (8q24), KCNQ3 (8q24), RNF139 (8q24), TNFRSF11B (8q24), FER1L6 (8q24.1), SQLE (8q24.1), EXT1 (8q24.11), MED30 (8q24.11), DEPTOR (8q24.12), DSCC1 (8q24.12), MTBP (8q24.12), SAMD12 (8q24.12), TAF2 (8q24.12), ANXA13 (8q24.13), FAM91A1 (8q24.13), FBXO32 (8q24.13), FER1L6-AS1 (8q24.13), KLHL38 (8q24.13), TATDN1 (8q24.13), TMEM65 (8q24.13), TRMT12 (8q24.13), ZNF572 (8q24.13), LRRC6 (8q24.22), OC90 (8q24.22), PHF20L1 (8q24.22), TMEM71 (8q24.22), INSM1 (20p11.2), RALGAPA2 (20p11.22) | 5 | (21.7) | 15 | (65.2) | 0.0133 |
| CRNKL1 (20p11.2), CST11 (20p11.21), CFAP61 (20p11.23), NAA20 (20p11.23) | 6 | (26.1) | 16 | (69.6) | 0.0133 |
| DEFB115 (20q11.21), FRG1BP (20q11.21) | 7 | (30.4) | 17 | (73.9) | 0.0133 |
| SAMHD1 (20pter-q12), EPPIN-WFDC6 (20q), ASXL1 (20q11), CBFA2T2 (20q11), NCOA6 (20q11), DEFB123 (20q11.1), DEFB124 (20q11.1), MAPRE1 (20q11.1-q11.23), BPIFA1 (20q11.2), CCM2L (20q11.2), DNMT3B (20q11.2), E2F1 (20q11.2), EIF2S2 (20q11.2), GHRH (20q11.2), GSS (20q11.2), MMP24 (20q11.2), RBL1 (20q11.2), BPIFA2 (20q11.21), BPIFA3 (20q11.21), BPIFB1 (20q11.21), BPIFB2 (20q11.21), BPIFB3 (20q11.21), BPIFB4 (20q11.21), BPIFB6 (20q11.21), C20orf203 (20q11.21), CDK5RAP1 (20q11.21), COMMD7 (20q11.21), DEFB118 (20q11.21), DEFB119 (20q11.21), DEFB121 (20q11.21), DYNLRB1 (20q11.21), EFCAB8 (20q11.21), HM13 (20q11.21), PDRG1 (20q11.21), REM1 (20q11.21), SUN5 (20q11.21), TM9SF4 (20q11.21), TTLL9 (20q11.21), XKR7 (20q11.21), NDRG3 (20q11.21-q11.23), RALY (20q11.21-q11.23), ACSS2 (20q11.22), ACTL10 (20q11.22), AHCY (20q11.22), C20orf144 (20q11.22), CHMP4B (20q11.22), EDEM2 (20q11.22), GGT7 (20q11.22), ITCH (20q11.22), MAP1LC3A (20q11.22), MROH8 (20q11.22), MYH7B (20q11.22), NECAB3 (20q11.22), PIGU (20q11.22), PXMP4 (20q11.22), TP53INP2 (20q11.22), TRPC4AP (20q11.22), UQCC1 (20q11.22), ZNF341 (20q11.22), DHX35 (20q11.22-q12), ACTR5 (20q11.23), ADIG (20q11.23), ARHGAP40 (20q11.23), BLCAP (20q11.23), DSN1 (20q11.23), FAM83D (20q11.23), MANBAL (20q11.23), MYL9 (20q11.23), PPP1R16B (20q11.23), RALGAPB (20q11.23), SLA2 (20q11.23), SLC32A1 (20q11.23), SOGA1 (20q11.23), TGIF2 (20q11.23), TLDC2 (20q11.23), CTNNBL1 (20q11.23-q12), ASIP (20q11.2-q12), STK4 (20q11.2-q13.2), HCK (20q11-q12), CHD6 (20q12), EMILIN3 (20q12), KCNS1 (20q12), LPIN3 (20q12), SDC4 (20q12), SLPI (20q12), ZHX3 (20q12), SRC (20q12-q13), PKIG (20q12-q13.1), PLCG1 (20q12-q13.1), RPN2 (20q12-q13.1), SEMG2 (20q12-q13.1), SRSF6 (20q12-q13.1), TOP1 (20q12-q13.1), TNNC2 (20q12-q13.11), CD40 (20q12-q13.2), SEMG1 (20q12-q13.2), CEBPB (20q13.1), CTSA (20q13.1), EYA2 (20q13.1), MYBL2 (20q13.1), YWHAB (20q13.1), ACOT8 (20q13.12), ADA (20q13.12), DBNDD2 (20q13.12), DNTTIP1 (20q13.12), EPPIN (20q13.12), FITM2 (20q13.12), GTSF1L (20q13.12), HNF4A (20q13.12), KCNK15 (20q13.12), LINC01620 (20q13.12), MATN4 (20q13.12), MMP9 (20q13.12), NCOA5 (20q13.12), NEURL2 (20q13.12), PCIF1 (20q13.12), PI3 (20q13.12), PIGT (20q13.12), PLTP (20q13.12), R3HDML (20q13.12), RBPJL (20q13.12), RIMS4 (20q13.12), SERINC3 (20q13.12), SLC12A5 (20q13.12), SNX21 (20q13.12), SPATA25 (20q13.12), SPINT3 (20q13.12), SPINT4 (20q13.12), SYS1 (20q13.12), SYS1-DBNDD2 (20q13.12), TP53TG5 (20q13.12), TTPAL (20q13.12), UBE2C (20q13.12), WFDC10A (20q13.12), WFDC10B (20q13.12), WFDC11 (20q13.12), WFDC12 (20q13.12), WFDC13 (20q13.12), WFDC2 (20q13.12), WFDC3 (20q13.12), WFDC5 (20q13.12), WFDC6 (20q13.12), WFDC8 (20q13.12), WFDC9 (20q13.12), WISP2 (20q13.12), ZMYND8 (20q13.12), ZNF335 (20q13.12), ZSWIM1 (20q13.12), ZSWIM3 (20q13.12), ARFGEF2 (20q13.13), LINC00494 (20q13.13), LINC01270 (20q13.13), PREX1 (20q13.13), SGK2 (20q13.2), TMEM189 (20q13.2), TMEM189-UBE2V1 (20q13.2), UBE2V1 (20q13.2) | 8 | (34.8) | 18 | (78.3) | 0.0133 |
| CYP24A1 (20q13), DOK5 (20q13.2), PFDN4 (20q13.2), APCDD1L (20q13.32) | 9 | (39.1) | 19 | (82.6) | 0.0133 |
| CDH4 (20q13.3), DNAJC5 (20q13.33), PCMTD2 (20q13.33), SAMD10 (20q13.33), TAF4 (20q13.33), UCKL1 (20q13.33), ZNF512B (20q13.33) | 10 | (43.5) | 20 | (87.0) | 0.0133 |
| ASIC3 (7q35), FASTK (7q35), ABCB8 (7q36), CDK5 (7q36), NOS3 (7q36), AGAP3 (7q36.1), ATG9B (7q36.1), GBX1 (7q36.1), SLC4A2 (7q36.1), TMUB1 (7q36.1), RP1 (8q12.1), TMEM68 (8q12.1), KLF10 (8q22.2), CHGB (20p12.3), MCM8 (20p12.3), TRMT6 (20p12.3), ADRA1D (20p13), ANGPT4 (20p13), CPXM1 (20p13), CSNK2A1 (20p13), FAM110A (20p13), MAVS (20p13), PANK2 (20p13), PCED1A (20p13), PRND (20p13), PRNP (20p13), PRNT (20p13), PTPRA (20p13), RBCK1 (20p13), RNF24 (20p13), RSPO4 (20p13), SCRT2 (20p13), SLC52A3 (20p13), SMOX (20p13), SRXN1 (20p13), STK35 (20p13), TBC1D20 (20p13), TCF15 (20p13), TMEM239 (20p13), VPS16 (20p13), TRIB3 (20p13-p12.2), ZCCHC3 (20p13-p12.2), CRLS1 (20p13-p12.3) | 3 | (13.0) | 13 | (56.5) | 0.0133-0.0486 |
| C7orf50 (7p22.3), GPER1 (7p22.3), ZFAND2A (7p22.3), OSR2 (8q22.2), VPS13B (8q22.2), ATP6V1C1 (8q22.3), ZNF706 (8q22.3), AGO2 (8q24), C8orf82 (8q24), FAM135B (8q24.23), COL22A1 (8q24.3), GPT (8q24.3), LRRC14 (8q24.3), MFSD3 (8q24.3), RECQL4 (8q24.3), DEFB125 (20p13), DEFB126 (20p13), DEFB127 (20p13) | 4 | (17.4) | 14 | (60.9) | 0.0133-0.0486 |
| NELFB (9q34), TRAF2 (9q34), PTGDS (9q34.2-q34.3), AGPAT2 (9q34.3), C8G (9q34.3), CCDC183 (9q34.3), CLIC3 (9q34.3), EDF1 (9q34.3), EGFL7 (9q34.3), FAM69B (9q34.3), FBXW5 (9q34.3), LCN10 (9q34.3), LCN12 (9q34.3), LCN15 (9q34.3), LCN6 (9q34.3), LCN8 (9q34.3), LCNL1 (9q34.3), MAMDC4 (9q34.3), PHPT1 (9q34.3), SNORA17A (9q34.3), TMEM141 (9q34.3) | 0 | (0.0) | 9 | (39.1) | 0.023 |
| TGS1 (8q11), TCEA1 (8q11.2), LYPLA1 (8q11.23), SOX17 (8q11.23), MRPL15 (8q11.2-q13), IMPAD1 (8q12.1), LYN (8q13), BMP2 (20p12), HAO1 (20p12), MKKS (20p12), JAG1 (20p12.1-p11.23), ANKEF1 (20p12.2), SLX4IP (20p12.2), SNAP25 (20p12-p11.2), GNRH2 (20p13), MRPS26 (20p13), OXT (20p13), RASSF2 (20p13), SLC23A2 (20p13) | 3 | (13.0) | 12 | (52.2) | 0.023 |
| PTPRT (20q12-q13), BMP7 (20q13), CSE1L (20q13), ADNP (20q13.13), BCAS4 (20q13.13), DPM1 (20q13.13), PARD6B (20q13.13), BCAS1 (20q13.2), TFAP2C (20q13.2), GNAS (20q13.3), CTCFL (20q13.31), FAM209B (20q13.31), PCK1 (20q13.31), ZBP1 (20q13.31), PMEPA1 (20q13.31-q13.33), ANKRD60 (20q13.32), C20orf85 (20q13.32), RAB22A (20q13.32), VAPB (20q13.33) | 9 | (39.1) | 18 | (78.3) | 0.023 |
| NBPF19 (1q21), NBPF20 (1q21.2), TERT (5p15.33), ABCA2 (9q34), ENTPD2 (9q34), MAN1B1 (9q34), C9orf139 (9q34.3), DPP7 (9q34.3), FUT7 (9q34.3), NPDC1 (9q34.3), SAPCD2 (9q34.3), UAP1L1 (9q34.3), RPS15A (16p), NOMO2 (16p12.3), ARL6IP1 (16p12-p11.2), SYNGR3 (16p13), FOPNL (16p13.11), MYH11 (16p13.11), NDE1 (16p13.11), NPIPA7 (16p13.11), BRICD5 (16p13.3), E4F1 (16p13.3), MLST8 (16p13.3), PGP (16p13.3), GFER (16p13.3-p13.12), COMP (19p13.1) | 1 | (4.3) | 10 | (43.5) | 0.023-0.0477 |
| HOXA13 (7p15.2), NPBWR1 (8p22-q21.13), RB1CC1 (8q11), SNTG1 (8q11.21), PXDNL (8q11.22-q11.23), PCMTD1 (8q11.23), PLAG1 (8q12), ASPH (8q12.1), CHCHD7 (8q12.1), XKR4 (8q12.1), CLVS1 (8q12.3), GGH (8q12.3), NKAIN3 (8q12.3), REXO1L2P (8q21.2), UBR5 (8q22), AZIN1 (8q22.3), ODF1 (8q22.3), RRM2B (8q23.1), KHDRBS3 (8q24.2), POU5F1B (8q24.21), CHRAC1 (8q24.3), TRAPPC9 (8q24.3) | 4 | (17.4) | 13 | (56.5) | 0.023-0.0477 |
| ZHX1-C8orf76 (8q), EYA1 (8q13.3), PTDSS1 (8q22), UQCRB (8q22), C8orf37 (8q22.1), GDF6 (8q22.1), MTERF3 (8q22.1), NCALD (8q22.2), PABPC1 (8q22.2-q23), GRHL2 (8q22.3), SNX31 (8q22.3), SDC2 (8q22-q23), EBAG9 (8q23), NUDCD1 (8q23), PKHD1L1 (8q23), TRHR (8q23), ENY2 (8q23.1), YWHAZ (8q23.1), KCNV1 (8q23.2), SYBU (8q23.2), CSMD3 (8q23.3), ADCY8 (8q24), SLA (8q24), TG (8q24), ENPP2 (8q24.1), NOV (8q24.1), EIF3H (8q24.11), TRPS1 (8q24.12), ATAD2 (8q24.13), C8orf76 (8q24.13), DERL1 (8q24.13), FAM83A (8q24.13), TBC1D31 (8q24.13), WDYHV1 (8q24.13), ZHX1 (8q24.13), ZHX2 (8q24.13), GSDMC (8q24.21), TMEM75 (8q24.21), EFR3A (8q24.22), DENND3 (8q24.3), KCNK9 (8q24.3), PTK2 (8q24.3), SLC45A4 (8q24.3), GGTLC1 (20p11.1), NANP (20p11.1), ZNF337 (20p11.1), APMAP (20p11.2), PCSK2 (20p11.2), ABHD12 (20p11.21), ACSS1 (20p11.21), CST1 (20p11.21), CST2 (20p11.21), CST3 (20p11.21), CST4 (20p11.21), CST5 (20p11.21), CST7 (20p11.21), ENTPD6 (20p11.21), GINS1 (20p11.21), SYNDIG1 (20p11.21), VSX1 (20p11.21), NINL (20p11.22-p11.1), KIZ (20p11.23), XRN2 (20p11.2-p11.1), BFSP1 (20p12.1) | 5 | (21.7) | 14 | (60.9) | 0.023-0.0477 |
| ZYX (7q32), KEL (7q33), EPHB6 (7q33-q35), EPHA1 (7q34), FAM131B (7q34), GSTK1 (7q34), OR6V1 (7q34), OR9A2 (7q34), PIP (7q34), PRSS58 (7q34), TAS2R39 (7q34), TAS2R40 (7q34), TMEM139 (7q34), TRPV6 (7q34), TRY2P (7q34), CASP2 (7q34-q35), CLCN1 (7q35), TAS2R41 (7q35), TAS2R60 (7q35), TRPV5 (7q35), LMBR1 (7q36), MRPL13 (8q22.1-q22.3), COL14A1 (8q23) | 6 | (26.1) | 15 | (65.2) | 0.023-0.0477 |
| BRAF (7q34), MRPS33 (7q34), TMEM178B (7q34), RIN2 (20p11.22) | 7 | (30.4) | 16 | (69.6) | 0.023-0.0477 |
| PROCR (20q11.2), SNTA1 (20q11.2), FAM83C (20q11.22), EIF6 (20q12), MAFB (20q12) | 8 | (34.8) | 17 | (73.9) | 0.023-0.0477 |
| BAIAP2 (17q25), AATK (17q25.3), CEP131 (17q25.3), CHMP6 (17q25.3), RPTOR (17q25.3), SLC38A10 (17q25.3) | 2 | (8.7) | 11 | (47.8) | 0.0477 |
| INHBB (2cen-q13), SIX3 (2p21), NPHP1 (2q13), GLI2 (2q14), EPB41L5 (2q14.2), RALB (2q14.2), TMEM185B (2q14.2), TFRC (3q29), CAPN11 (6p12), HSP90AB1 (6p12), VEGFA (6p12), C6orf223 (6p21.1), NFKBIE (6p21.1), SLC29A1 (6p21.1), SLC35B2 (6p21.1), TCTE1 (6p21.1), TMEM151B (6p21.1), TMEM63B (6p21.1), MRPL14 (6p21.3), CD19 (16p11.2), LAT (16p11.2), NFATC2IP (16p11.2), RABEP2 (16p11.2), SH2B1 (16p11.2), SPNS1 (16p11.2), ATP2A1 (16p12.1), SNN (16p13), LITAF (16p13.13), TXNDC11 (16p13.13) | 0 | (0.0) | 8 | (34.8) | 0.0399 |
| SKI (1p36.33), NBPF9 (1q21.1), RUSC2 (9p13.3), NOTCH1 (9q34.3), FAHD1 (16p13.3), HAGH (16p13.3), HS3ST6 (16p13.3), MEIOB (16p13.3), MSRB1 (16p13.3), NDUFB10 (16p13.3), NOXO1 (16p13.3), RNF151 (16p13.3), RPL3L (16p13.3), RPS2 (16p13.3), TBL3 (16p13.3), UPF1 (19p13.11) | 1 | (4.3) | 9 | (39.1) | 0.0399 |
| CEBPD (8p11.2-p11.1), PRKDC (8q11), MCM4 (8q11.2), SPIDR (8q11.21), UBE2V2 (8q11.21), ARFGEF1 (8q13), ARMC1 (8q13.1), COPS5 (8q13.1), MCMDC2 (8q13.1), MTFR1 (8q13.1), PPP1R42 (8q13.1), TCF24 (8q13.1), CSPP1 (8q13.2), NRSN2 (20p13), SOX12 (20p13) | 3 | (13.0) | 11 | (47.8) | 0.0399 |
| ATP6V1H (8q11.2), RGS20 (8q11.23), ST18 (8q11.23), CYP7A1 (8q11-q12), SDCBP (8q12), SDR16C5 (8q12.1), TOX (8q12.1), UBXN2B (8q12.1), NSMAF (8q12-q13), CA3 (8q21.2), CALB1 (8q21.3), DECR1 (8q21.3), PSKH2 (8q21.3), SLC7A13 (8q21.3), CA2 (8q22), TP53INP1 (8q22), CCNE2 (8q22.1), CDH17 (8q22.1), DPY19L4 (8q22.1), ESRP1 (8q22.1), INTS8 (8q22.1), PDP1 (8q22.1), PENK (8q23-q24), FAM84B (8q24.21), PLCB1 (20p12) | 4 | (17.4) | 12 | (52.2) | 0.0399 |
| CPA6 (8q13.2), KCNB2 (8q13.2), LACTB2 (8q13.3), NCOA2 (8q13.3), PRDM14 (8q13.3), TRAM1 (8q13.3), XKR9 (8q13.3), ZBTB10 (8q13-q21.1), FABP4 (8q21), WWP1 (8q21), LY96 (8q21.11), SBSPON (8q21.11), STAU2 (8q21.11), TERF1 (8q21.11), TMEM70 (8q21.11), UBE2W (8q21.11), FABP12 (8q21.13), FABP5 (8q21.13), FABP9 (8q21.13), PAG1 (8q21.13), ZNF704 (8q21.13), CNGB3 (8q21.3), CPNE3 (8q21.3), RMDN1 (8q21.3), PMP2 (8q21.3-q22.1), C8orf87 (8q22.1), RBM12B (8q22.1), TRIQK (8q22.1), ANKRD46 (8q22.2), EIF3E (8q22-q23), ANGPT1 (8q23.1), EMC2 (8q23.1), RSPO2 (8q23.1), RAD21 (8q24), AARD (8q24.11), SLC30A8 (8q24.11), UTP23 (8q24.11), HAS2 (8q24.12), NSMCE2 (8q24.13), TRIB1 (8q24.13), ASAP1 (8q24.1-q24.2), FAM49B (8q24.21), ZFAT (8q24.22), RBBP9 (20p11.2), C20orf78 (20p11.23), DTD1 (20p11.23), SCP2D1 (20p11.23), SEC23B (20p11.23), LAMP5 (20p12), PLCB4 (20p12), RRBP1 (20p12), BANF2 (20p12.1), DSTN (20p12.1) | 5 | (21.7) | 13 | (56.5) | 0.0399 |
| CST8 (20p11.21), CST9 (20p11.21), CST9L (20p11.21) | 6 | (26.1) | 14 | (60.9) | 0.0399 |
| AURKA (20q13), KCNG1 (20q13), MOCS3 (20q13.13), ATP9A (20q13.2), CSTF1 (20q13.2), FAM210B (20q13.2), GCNT7 (20q13.2), NFATC2 (20q13.2), SALL4 (20q13.2), ZFP64 (20q13.2), ZNF217 (20q13.2), CASS4 (20q13.31), FAM209A (20q13.31) | 9 | (39.1) | 17 | (73.9) | 0.0399 |
| PTPN1 (20q13.1-q13.2) | 10 | (43.5) | 18 | (78.3) | 0.0399 |
| **LOH** |  |  |  |  |  |
| PDGFRL (8p22-p21.3), SLC7A2 (8p22) | 1 | (4.3) | 9 | (39.1) | 0.0399 |
| CTC1 (17p13.1), PFAS (17p13.1) | 3 | (13.0) | 12 | (52.2) | 0.0477 |
| **CN-LOH** |  |  |  |  |  |
| C17orf75 (17q11.2), CDK5R1 (17q11.2), PSMD11 (17q11.2), RHBDL3 (17q11.2), ZNF207 (17q11.2), RPL3 (22q13), MGAT3 (22q13.1), SYNGR1 (22q13.1), TAB1 (22q13.1) | 0 | (0.0) | 8 | (34.8) | 0.0399 |

HGIL, high-grade intramucosal lesion; ISL, invasive submucosal lesion; LOH, loss of heterozygosity; CN-LOH, copy neutral loss of heterozygosity.

Supplementary Table 5. Somatic copy number alterations with significant differences between the invasive submucosal lesions and invasive front lesions (1146 genes with gains, one gene with LOH).

| Genes (locus) | ISL positive (%) | | IFL positive (%) | | *p* - value |
| --- | --- | --- | --- | --- | --- |
| **Gain** |  |  |  |  |  |
| FAM72B (1p11.2), FCGR1B (1p11.2), SRGAP2C (1p11.2), FAM72D (1q21.1), PPIAL4G (1q21.1), SRGAP2B (1q21.1) | 1 | (4.3) | 12 | (52.2) | 0.0051 |
| FAM231D (1q21.2), NBPF14 (1q21.2), NBPF15 (1q21.2), PPIAL4E (1q21.2), PPIAL4F (1q21.2), MYCN (2p24.3), OSTN (3q28), ZNF71 (19q13.4), ERVV-1 (19q13.41), ZNF350 (19q13.41), ZNF432 (19q13.41), ZNF613 (19q13.41), ZNF614 (19q13.41), ZNF615 (19q13.41), ZNF841 (19q13.41), VN1R2 (19q13.42), VN1R4 (19q13.42), ZNF160 (19q13.42), ZNF347 (19q13.42), ZNF415 (19q13.42), ZNF525 (19q13.42), ZNF665 (19q13.42), ZNF677 (19q13.42), ZNF845 (19q13.42), ZNF470 (19q13.43), ZNF471 (19q13.43) | 2 | (8.7) | 12 | (52.2) | 0.0133 |
| EML6 (2p16.1), SPTBN1 (2p21) | 5 | (21.7) | 15 | (65.2) | 0.0133 |
| LRCH1 (13q14.11), LRRC63 (13q14.13), ESD (13q14.1-q14.2), RB1 (13q14.2), CYSLTR2 (13q14.2) | 8 | (34.8) | 18 | (78.3) | 0.0133 |
| KBTBD2 (7p14.3), KL (13q12), FOXO1 (13q14.1), ITGBL1 (13q33) | 9 | (39.1) | 19 | (82.6) | 0.0133 |
| CDK13 (7p13), MPLKIP (7p14.1), SUGCT (7p14.1), AGMO (7p21.2), TMEM106B (7p21.3), VWDE (7p21.3), ARL4A (7p21.3), SCIN (7p21.3), MEOX2 (7p22.1-p21.3) | 11 | (47.8) | 21 | (91.3) | 0.0133 |
| SKAP2 (7p15.2) | 12 | (52.2) | 22 | (95.7) | 0.0133 |
| NBPF8 (1q21.1), PPIAL4A (1q21.1), PPIAL4C (1q21.2), CCDC138 (2q12.3), GCC2 (2q12.3), LIMS1 (2q12.3), RANBP2 (2q12.3), SULT1C4 (2q12.3), EDAR (2q13), RGPD3 (2q13), FAP (2q23), IFIH1 (2q24) | 3 | (13) | 13 | (56.5) | 0.0133-0.0281 |
| PDE4DIP (1q12), RPRM (2q23.3), TBR1 (2q24), PSMD14 (2q24.2), TANK (2q24.2), COBLL1 (2q24.3), CSRNP3 (2q24.3), SCN1A (2q24.3), RFPL4A (19q13.42), RFPL4AL1 (19q13.42) | 4 | (17.4) | 14 | (60.9) | 0.0133-0.0281 |
| NT5C3A (7p14.3), RP9 (7p14.3), ANKMY2 (7p21), BZW2 (7p21.1), ITGB8 (7p21.1), LRRC72 (7p21.1), SOSTDC1 (7p21.1), TSPAN13 (7p21.1), AGR2 (7p21.3), CD36 (7q11.2), ADAM22 (7q21), HGF (7q21.1), PCLO (7q21.11), GNAT3 (7q21.11), SEMA3E (7q21.11), ABCB1 (7q21.12), RUNDC3B (7q21.12), SLC25A40 (7q21.12), ANKIB1 (7q21.2), FAM133B (7q21.2), PEX1 (7q21.2), RBM48 (7q21.2), DBF4 (7q21.3), GATAD1 (7q21-q22), SEMA3C (7q21-q31), FAM185A (7q22.1), TAS2R16 (7q31.1-q31.3), ASB15 (7q31.31), IQUB (7q31.32), LMOD2 (7q31.32), SLC13A1 (7q31.32), NDUFA5 (7q31.33), GRM8 (7q31.3-q32.1), KLF12 (13q22) | 10 | (43.5) | 20 | (87) | 0.0133-0.0281 |
| CYP2J2 (1p31.3-p31.2), FGGY (1p32.1), HOOK1 (1p32.1), C1orf87 (1p32.1), AGO4 (1p34) | 0 | (0) | 9 | (39.1) | 0.0153-0.023 |
| FZD8 (10p11.21), GJD4 (10p11.21), MTRNR2L7 (10p11.21), SLC22A10 (11q12.3), SLC22A24 (11q12.3), SLC22A25 (11q12.3), SLC22A9 (11q13.1), CAPRIN2 (12p11), IPO8 (12p11.21), DIP2B (12q13.12) | 1 | (4.3) | 10 | (43.5) | 0.023 |
| PRR20B (13q21.1), PRR20C (13q21.1), PRR20D (13q21.1) | 7 | (30.4) | 16 | (69.6) | 0.023 |
| LPAR6 (13q14), MED4 (13q14.2), NUDT15 (13q14.2), SUCLA2 (13q14.2), ITM2B (13q14.3), RCBTB2 (13q14.3) | 8 | (34.8) | 17 | (73.9) | 0.023 |
| C7orf71 (7p15.2), ISPD (7p21.2), DGKB (7p21.2) | 12 | (52.2) | 21 | (91.3) | 0.023 |
| NOTCH2 (1p13-p11), CEP170 (1q44), KCNMB3 (3q26.3-q27), KHDRBS2 (6q11.1), MTRNR2L9 (6q11.1), PHF3 (6q12), EYS (6q12), CD109 (6q13), BCKDHB (6q14.1), FILIP1 (6q14.1), HMGN3 (6q14.1), LCA5 (6q14.1), SENP6 (6q14.1), SH3BGRL2 (6q14.1), TMEM30A (6q14.1), KIAA1551 (12p11.21), BICD1 (12p11.2-p11.1), C12orf29 (12q21.32), C12orf50 (12q21.32), CEP290 (12q21.32), ZNF146 (19q13.1), ZNF565 (19q13.12), PEG3 (19q13.4), PPP2R1A (19q13.41), ZNF480 (19q13.41), ZNF616 (19q13.41), ZNF766 (19q13.41), ZNF836 (19q13.41), USP29 (19q13.43), SMIM17 (19q13.43), ZNF582 (19q13.43), ZNF583 (19q13.43), ZNF667 (19q13.43), ZNF835 (19q13.43) | 2 | (8.7) | 11 | (47.8) | 0.023-0.0477 |
| ARID4B (1q42.3), GGPS1 (1q43), FANCL (2p16.1), QPCT (2p22.2), CMPK2 (2p25.2), RSAD2 (2p25.2), DPP4 (2q24.3), GCG (2q36-q37), PIK3CA (3q26.3), KCNMB2 (3q26.32), CCDC50 (3q28), UTS2B (3q28), DUSP22 (6p25.3), USP15 (12q14), FAM19A2 (12q14.1), MON2 (12q14.1), SHCBP1 (16q11.2), RPSAP58 (19p12), ZNF675 (19p12), ZNF681 (19p12), ZNF726 (19p12), ZNF728 (19p12), ZNF730 (19p12), ZNF91 (19p12), ZNF99 (19p12), ZNF816-ZNF321P (19q), THEG5 (19q12), ZNF528 (19q13), CLC (19q13.1), FBL (19q13.1), FCGBP (19q13.1), DYRK1B (19q13.2), LEUTX (19q13.2), LGALS14 (19q13.2), LGALS17A (19q13.2), ZNF112 (19q13.2), ZNF221 (19q13.2), ZNF222 (19q13.2), ZNF223 (19q13.2), ZNF224 (19q13.2), ZNF225 (19q13.2), ZNF226 (19q13.2), ZNF235 (19q13.2), ZNF45 (19q13.2), ZNF155 (19q13.2-q13.32), KLK6 (19q13.3), LYPD5 (19q13.31), ZNF230 (19q13.31), ZNF233 (19q13.31), ZNF234 (19q13.31), ZNF283 (19q13.31), ZNF284 (19q13.31), ZNF404 (19q13.31), CCDC9 (19q13.32), INAFM1 (19q13.32), SAE1 (19q13.32), KLK5 (19q13.33), BBC3 (19q13.3-q13.4), FPR2 (19q13.3-q13.4), FPR3 (19q13.3-q13.4), KLK2 (19q13.41), KLK4 (19q13.41), KLK7 (19q13.41), ZNF321P (19q13.41), ZNF534 (19q13.41), ZNF577 (19q13.41), ZNF610 (19q13.41), ZNF649 (19q13.41), ZNF816 (19q13.41), ZNF880 (19q13.41), NLRP4 (19q13.43) | 3 | (13) | 12 | (52.2) | 0.023-0.0477 |
| SEC22B (1q21.1), MDH1 (2p13.3), REL (2p13-p12), PNPT1 (2p15), WDPCP (2p15), EFEMP1 (2p16), CCDC85A (2p16.1), CFAP36 (2p16.1), PPP4R3B (2p16.1), VRK2 (2p16.1), FSHR (2p21-p16), THUMPD2 (2p22.1), FAM49A (2p24.2), NT5C1B (2p24.2), FAM84A (2p24.3), TRIB2 (2p24.3), FAM110C (2p25.3), C2orf49 (2q12.1), FHL2 (2q12.2), ST6GAL2 (2q12.3), SULT1C2 (2q12.3), SULT1C3 (2q12.3), FBLN7 (2q13), RGPD8 (2q13), TMEM87B (2q13), ZC3H6 (2q13), ZC3H8 (2q13), MERTK (2q14.1), CXCR4 (2q21), NXPH2 (2q22.1), SPOPL (2q22.1), ACVR2A (2q22.3), ORC4 (2q22-q23), MBD5 (2q23.1), ERMN (2q24.1), GALNT5 (2q24.1), GCA (2q24.2), TTC21B (2q24.3), GALNT3 (2q24-q31), NOSTRIN (2q31.1), TFPI (2q32), CALCRL (2q32.1), ZNF148 (3q21), SNX4 (3q21.2), ANAPC13 (3q22.2), CEP63 (3q22.2), KY (3q22.2), SLC25A36 (3q23), EIF5A2 (3q26.2), RPL22L1 (3q26.2), RTP4 (3q27.3), TMEM159 (16p12), ZP2 (16p12), ANKS4B (16p12.2), ERCC4 (16p13.12), TSHZ3 (19q12), ZNF83 (19q13.3), ZNF28 (19q13.41), ZNF600 (19q13.41), ZNF611 (19q13.41), ZNF701 (19q13.41), ZNF808 (19q13.41), NLRP11 (19q13.43) | 4 | (17.4) | 13 | (56.5) | 0.023-0.0477 |
| ACYP2 (2p16.2), C2orf73 (2p16.2), TSPYL6 (2p16.2), RTN4 (2p16.3), ROCK2 (2p24), ASNSD1 (2p24.3-q21.3), C2orf50 (2p25.1), E2F6 (2p25.1), FLJ33534 (2p25.1), GREB1 (2p25.1), LPIN1 (2p25.1), NTSR2 (2p25.1), PQLC3 (2p25.1), MAP4K4 (2q11.2-q12), IL1R2 (2q12), UXS1 (2q12.2), DPP10 (2q14.1), COL5A2 (2q14-q32), THSD7B (2q22.1), GRB14 (2q22-q24), LYPD6 (2q23.2), MMADHC (2q23.2), PLA2R1 (2q23-q24), SCN3A (2q24), CCDC148 (2q24.1), DAPL1 (2q24.1), GALNT13 (2q24.1), KCNJ3 (2q24.1), PKP4 (2q24.1), UPP2 (2q24.1), ITGB6 (2q24.2), KCNH7 (2q24.2), SLC4A10 (2q24.2), TANC1 (2q24.2), FIGN (2q24.3), SCN2A (2q24.3), SLC38A11 (2q24.3), FASTKD1 (2q31), BBS5 (2q31.1), CCDC173 (2q31.1), KLHL41 (2q31.1), PMS1 (2q31.1), PPIG (2q31.1), ORMDL1 (2q32), SLC40A1 (2q32), ANKAR (2q32.2), OSGEPL1 (2q32.2), WDR75 (2q32.2) | 5 | (21.7) | 14 | (60.9) | 0.023-0.0477 |
| PON2 (7q21.3), RASA4 (7q22), POLR2J3 (7q22.1), SPDYE2 (7q22.1), SPDYE2B (7q22.1), FRY (13q13.1), RXFP2 (13q13.1), ZAR1L (13q13.1), SLC25A15 (13q14), CPB2 (13q14.11), MRPS31 (13q14.11), ZC3H13 (13q14.13), LCP1 (13q14.3), HTR2A (13q14-q21), PCDH17 (13q21.1), LINC00550 (13q21.33), COMMD6 (13q22), EDNRB (13q22), LMO7 (13q22.2), LMO7DN (13q22.2), TBC1D4 (13q22.2), UCHL3 (13q22.2), SLAIN1 (13q22.3) | 9 | (39.1) | 18 | (78.3) | 0.023-0.0477 |
| FKBP9 (7p11.1), SEMA3A (7p12.1), NXPH1 (7p22), DMTF1 (7q21), GNAI1 (7q21), ABCB4 (7q21.1), CROT (7q21.1), SRI (7q21.1), SEMA3D (7q21.11), KIAA1324L (7q21.12), STEAP4 (7q21.12), TMEM243 (7q21.12), KRIT1 (7q21.2), EFCAB10 (7q22.3), PUS7 (7q22.3), RINT1 (7q22.3), SRPK2 (7q22-q31.1), FOXP2 (7q31), WNT2 (7q31.2), LEP (7q31.3), WASL (7q31.3), PTPRZ1 (7q31.3), MGC27345 (7q32.1), RBM28 (7q32.1), FLT1 (13q12), EPSTI1 (13q13.3), DNAJC15 (13q14.1), CCDC122 (13q14.11), ENOX1 (13q14.11), LACC1 (13q14.11), FAM124A (13q14.3), INTS6 (13q14.3), SERPINE3 (13q14.3), DLEU7 (13q14.3), RNASEH2B (13q14.3), DIAPH3 (13q21.2), PCDH9 (13q21.32), KLF5 (13q22.1), BORA (13q22.1), DIS3 (13q22.1), MZT1 (13q22.1), PIBF1 (13q22.1), KCTD12 (13q22.3), PCCA (13q32), GPC6 (13q32), GGACT (13q32.3), TMTC4 (13q32.3), EFNB2 (13q33), ARGLU1 (13q33.3), TEX29 (13q34), FGF14 (13q34) | 10 | (43.5) | 19 | (82.6) | 0.023-0.0477 |
| GLI3 (7p13), BBS9 (7p14), ELMO1 (7p14.1), EPDR1 (7p14.1), GPR141 (7p14.1), NME8 (7p14.1), POU6F2 (7p14.1), SFRP4 (7p14.1), DPY19L1 (7p14.2), BMPER (7p14.3), FKBP14 (7p14.3), NEUROD6 (7p14.3), NPSR1 (7p14.3), PRR15 (7p14.3), SCRN1 (7p14.3), WIPF3 (7p14.3), AMPH (7p14-p13), VPS41 (7p14-p13), HNRNPA2B1 (7p15), AHR (7p15), CBX3 (7p15.2), NFE2L3 (7p15.2), SNX10 (7p15.2), C7orf31 (7p15.3), CDCA7L (7p15.3), CYCS (7p15.3), NPVF (7p15.3), RAPGEF5 (7p15.3), SP4 (7p15.3), INHBA (7p15-p13), RALA (7p15-p13), DNAH11 (7p21), AGR3 (7p21.1), PRPS1L1 (7p21.1), SNX13 (7p21.1), COL28A1 (7p21.3), MIOS (7p21.3), NDUFA4 (7p21.3), PHF14 (7p21.3), THSD7A (7p21.3), UMAD1 (7p21.3), AIMP2 (7p22), ANKRD61 (7p22), EIF2AK1 (7p22), RPA3 (7p22), CYTH3 (7p22.1), USP42 (7p22.1), MAGI2 (7q21), STEAP2 (7q21.13), ZNF804B (7q21.13), CDK6 (7q21-q22), AKAP9 (7q21-q22), RELN (7q22), ARMC10 (7q22.1), FBXL13 (7q22.1), LRRC17 (7q22.1), NAPEPLD (7q22.1), CADPS2 (7q31.3), CHRM2 (7q31-q35), STARD13 (13q13.1) | 11 | (47.8) | 20 | (87) | 0.023-0.0477 |
| WARS2 (1p12), PRKAA2 (1p31), SCP2 (1p32), TAL1 (1p32), STIL (1p32), C8B (1p32.2), PLPP3 (1p32.2), COA7 (1p32.3), ECHDC2 (1p32.3), ZYG11A (1p32.3), ZYG11B (1p32.3), ZMYM4 (1p32-p34), ATPAF1 (1p33), EFCAB14 (1p33), MOB3C (1p33), TEX38 (1p33), CYP4A11 (1p33), CYP4A22 (1p33), CYP4B1 (1p33), CYP4X1 (1p33), CYP4Z1 (1p33), PDZK1IP1 (1p33), UROD (1p34), AGO3 (1p34), HECTD3 (1p34.1), KIAA0319L (1p34.2), PSMB2 (1p34.2), CLSPN (1p34.2), SH3D21 (1p34.3), C1orf216 (1p34.3), NCDN (1p34.3), TEKT2 (1p34.3), TFAP2E (1p34.3), AGO1 (1p34.3), IPP (1p34-p32), C5orf46 (5q32), DPYSL3 (5q32), JAKMIP2 (5q32), JAKMIP2-AS1 (5q32) | 0 | (0) | 8 | (34.8) | 0.0266-0.0399 |
| NBPF7 (1p12), SPINK5 (5q32), ADK (10q22), SLC22A8 (11q11), GXYLT1 (12q12), PPHLN1 (12q12), PRICKLE1 (12q12), YAF2 (12q12), ZCRB1 (12q12), CLLU1 (12q22), CLLU1OS (12q22) | 1 | (4.3) | 9 | (39.1) | 0.0399 |
| LY75-CD302 (2q), LGSN (6pter-q22.33), ADGRB3 (6q12), COX7A2 (6q12), LOC441155 (6q12), PTP4A1 (6q12), COL12A1 (6q12-q13), COL19A1 (6q12-q13), B3GAT2 (6q13), COL9A1 (6q13), DDX43 (6q13), DPPA5 (6q13), FAM135A (6q13), HTR1B (6q13), KHDC1 (6q13), KHDC3L (6q13), MTO1 (6q13), MYO6 (6q13), OOEP (6q13), SDHAF4 (6q13), SLC17A5 (6q13), SMAP1 (6q13), ELOVL4 (6q14), PHIP (6q14), EEF1A1 (6q14.1), IBTK (6q14.1), TTK (6q14.1), UBE3D (6q14.1), IMPG1 (6q14.2-q15), IRAK1BP1 (6q14-q15), TPBG (6q14-q15), EPHA7 (6q16.1), FOXO3 (6q21), ZNF248 (10p11.2), MASTL (10p12.1), TMEM236 (10p12.33), OR5AN1 (11q12.1), OR5AR1 (11q12.1), OR5T1 (11q12.1), OR8H1 (11q12.1), OR8K1 (11q12.1), OR8K3 (11q12.1), OR9G1 (11q12.1), OR9G4 (11q12.1), HRASLS2 (11q12.3), PLA2G16 (11q12.3), LGALS12 (11q13), UCP2 (11q13), ATL3 (11q13.1), HRASLS5 (11q13.2), RAB6A (11q13.3), COA4 (11q13.4), DNAJB13 (11q13.4), FAM168A (11q13.4), MRPL48 (11q13.4), PAAF1 (11q13.4), UCP3 (11q13.4), PLEKHB1 (11q13.5-q14.1), RARRES3 (11q23), OR10G4 (11q24.1), OR10S1 (11q24.1), OR4D5 (11q24.1), OR6M1 (11q24.1), OR6T1 (11q24.1), OR8D4 (11q24.1), TMEM225 (11q24.1), ARHGAP32 (11q24.3), BARX2 (11q25), PKP2 (12p11), ITPR2 (12p11), SYT10 (12p11.1), SSPN (12p11.2), DNM1L (12p11.21), FGD4 (12p11.21), YARS2 (12p11.21), CCDC91 (12p11.22), ERGIC2 (12p11.22), FAR2 (12p11.22), OVCH1 (12p11.22), TMTC1 (12p11.22), BHLHE41 (12p12.1), LMO3 (12p12.3), RASSF8 (12p12.3), STRAP (12p12.3), ALG10B (12q12), ATF1 (12q13), METTL7A (12q13.12), TMPRSS12 (12q13.12), NUDT4 (12q21), EPYC (12q21), ATP2B1 (12q21.3), TMTC3 (12q21.32), CCER1 (12q21.33), DCN (12q21.33), LINC00615 (12q21.33), LUM (12q21.33), POC1B (12q21.33), CEP83 (12q22), FGD6 (12q22), METAP2 (12q22), NR2C1 (12q22), NTN4 (12q22), TMPO (12q22), USP44 (12q22), VEZT (12q22), KERA (12q22), KITLG (12q22), NDUFA12 (12q22), TMCC3 (12q22), HAL (12q22-q24.1), APAF1 (12q23), SLC25A3 (12q23), AMDHD1 (12q23.1), CCDC38 (12q23.1), IKBIP (12q23.1), SNRPF (12q23.1), PLXNC1 (12q23.3), HBA1 (16p13.3), HBA2 (16p13.3), HBM (16p13.3), HBQ1 (16p13.3), HBZ (16p13.3), LUC7L (16p13.3), POLR2I (19q12), COX7A1 (19q13.1), SLC7A10 (19q13.1), LRP3 (19q13.11), TBCB (19q13.11-q13.12), CAPNS1 (19q13.12), OVOL3 (19q13.12), LILRA6 (19q13.4), LILRB2 (19q13.4), LILRB5 (19q13.4) | 2 | (8.7) | 10 | (43.5) | 0.0399 |
| ASPM (1q31), B3GALT2 (1q31), RGS1 (1q31), RGS2 (1q31), TROVE2 (1q31), GLRX2 (1q31.2), RGS13 (1q31.2), RGS18 (1q31.2), RGS21 (1q31.2), ATP6V1G3 (1q31.3), CFHR2 (1q31.3), CFHR5 (1q31.3), KCNT2 (1q31.3), PTPRC (1q31-q32), F13B (1q31-q32.1), CD46 (1q32), CFH (1q32), CFHR1 (1q32), CFHR3 (1q32), CFHR4 (1q32), UCHL5 (1q32), CR1L (1q32.1), LYPLAL1 (1q41), BPNT1 (1q41), C1orf115 (1q41), EPRS (1q41), HLX (1q41), IARS2 (1q41), MARK1 (1q41), RAB3GAP2 (1q41), SLC30A10 (1q41), FH (1q42.1), CNIH3 (1q42.12), KMO (1q42-q44), BECN2 (1q43), CHML (1q43), MAP1LC3C (1q43), OPN3 (1q43), PLD5 (1q43), SDCCAG8 (1q43), FOXI3 (2p11.2), RPIA (2p11.2), TEX37 (2p11.2), EIF2AK3 (2p12), BMP10 (2p13.3), GKN1 (2p13.3), GKN2 (2p13.3), MEIS1 (2p14), RPS27A (2p16), CLHC1 (2p16.1), MTIF2 (2p16.1), CDC42EP3 (2p21), SRD5A2 (2p23), TEKT4 (2q11.1), NEB (2q22), NMI (2q23), ARL6IP6 (2q23.3), PRPF40A (2q23.3), RIF1 (2q23.3), TNFAIP6 (2q23.3), P2RY1 (3q25.2), ZMAT3 (3q26.32), FAM83B (6p12.1), PRIM2 (6p12-p11.1), RNF217 (6q22.31), EYA4 (6q23), SGK1 (6q23), SLC2A12 (6q23.2), TBPL1 (6q23.2), TCF21 (6q23.2), AHI1 (6q23.3), HBS1L (6q23.3), PDE7B (6q23-q24), UTRN (6q24), NEBL (10p12), TRIM48 (11q11), AHNAK (11q12.2), ASRGL1 (11q12.3), EEF1G (11q12.3), SCGB1A1 (11q12.3), SCGB1D4 (11q12.3), SCGB2A2 (11q13), EPS8 (12p12.3), CPNE8 (12q12), DBX2 (12q12), NELL2 (12q12), TMEM117 (12q12), SLC16A7 (12q13), LINC01465 (12q14.1), LRIG3 (12q14.1), PPM1H (12q14.1), TSPAN8 (12q14.1-q21.1), AVPR1A (12q14.2), IRAK3 (12q14.3), LLPH (12q14.3), MSRB3 (12q14.3), TMBIM4 (12q14.3), MYRFL (12q15), PTPRR (12q15), RAB3IP (12q15), PTPRB (12q15-q21), TRHDE (12q15-q21), RAB21 (12q21.1), THAP2 (12q21.1), TMEM19 (12q21.1), TPH2 (12q21.1), ZFC3H1 (12q21.1), LGR5 (12q22-q23), ZNF737 (19p12), ZNF93 (19p12), OR7C2 (19p13.1), ZNF253 (19p13.11), ZNF506 (19p13.11), OR7A17 (19p13.12), SLC1A6 (19p13.12), KLK10 (19q13), KLK8 (19q13), AXL (19q13.1), CYP2S1 (19q13.1), GPI (19q13.1), SLC7A9 (19q13.1), GPATCH1 (19q13.11), WDR88 (19q13.11), ANKRD27 (19q13.11), CEP89 (19q13.11), FAAP24 (19q13.11), KIAA0355 (19q13.11), LSM14A (19q13.11), NUDT19 (19q13.11), PDCD2L (19q13.11), PDCD5 (19q13.11), RGS9BP (19q13.11), RHPN2 (19q13.11), TDRD12 (19q13.11), ZFP14 (19q13.12), CYP2A7 (19q13.2), CYP2A13 (19q13.2), CYP2B6 (19q13.2), CYP2F1 (19q13.2), HNRNPUL1 (19q13.2), CALM3 (19q13.2-q13.3), LIG1 (19q13.2-q13.3), CD33 (19q13.3), EMP3 (19q13.3), ETFB (19q13.3), SIGLEC7 (19q13.3), CARD8 (19q13.33), CCDC114 (19q13.33), KLK11 (19q13.33), KLK12 (19q13.33), KLK13 (19q13.33), MYH14 (19q13.33), TMEM143 (19q13.33), ZNF114 (19q13.33), KLK14 (19q13.3-q13.4), LIM2 (19q13.4), ZNF304 (19q13.4), C19orf84 (19q13.41), CLDND2 (19q13.41), CTU1 (19q13.41), IGLON5 (19q13.41), KLK3 (19q13.41), KLK9 (19q13.41), NKG7 (19q13.41), SIGLEC9 (19q13.41), SIGLECL1 (19q13.41), VSIG10L (19q13.41), ZNF888 (19q13.41), ZNF543 (19q13.43), ZNF547 (19q13.43), ZNF548 (19q13.43) | 3 | (13) | 11 | (47.8) | 0.0399 |
| OTX1 (2p13), PROKR1 (2p13.1), APLF (2p13.3), ARHGAP25 (2p13.3), FBXO48 (2p13.3), PLEK (2p13.3), C1D (2p13-p12), CNRIP1 (2p14), ETAA1 (2p14), PNO1 (2p14), WDR92 (2p14), C2orf74 (2p15), EHBP1 (2p15), LOC339803 (2p15), PPP3R1 (2p15), USP34 (2p15), XPO1 (2p15), PEX13 (2p16.1), PUS10 (2p16.1), KIAA1841 (2q14), SCN7A (2q21-q23), SCN9A (2q24), SPC25 (2q31.1), CRYGC (2q33.3), CRYGD (2q33.3), CRYGA (2q34), CRYGB (2q34), LANCL1 (2q34), OSBPL11 (3q21), CHST2 (3q24), EIF2A (3q25.1), MED12L (3q25.1), P2RY12 (3q25.1), SERP1 (3q25.1), C3orf33 (3q25.31), SERPINI2 (3q26.1), WDR49 (3q26.1), ZBBX (3q26.1), GMNC (3q28), PYDC2 (3q28), LRRC1 (6p12.1), MLIP (6p12.1), TINAG (6p12.1), B4GALNT3 (12p13.33), ZNF536 (19q12), LGALS13 (19q13.1), EID2 (19q13.2), LGALS16 (19q13.2), ZNF180 (19q13.2), ZNF229 (19q13.31), ZNF154 (19q13.4), ZNF320 (19q13.41), ZNF468 (19q13.41), ZNF578 (19q13.41), ZNF761 (19q13.42), ZNF765 (19q13.42), ZNF813 (19q13.42), DPRX (19q13.42), ZNF331 (19q13.42), ZNF551 (19q13.43), ZSCAN4 (19q13.43), NLRP13 (19q13.43), NLRP8 (19q13.43) | 4 | (17.4) | 12 | (52.2) | 0.0399 |
| KIDINS220 (2p24), MBOAT2 (2p25.1), C2orf40 (2q12.2), DNAH7 (2q32.3), SLC39A10 (2q32.3), STK17B (2q32.3), AOX1 (2q33), KCTD18 (2q33.1), SPATS2L (2q33.1), CARF (2q33.2), CYP20A1 (2q33.2), ICA1L (2q33.2), NBEAL1 (2q33.2), WDR12 (2q33.2), PCOLCE2 (3q21-q24), CDV3 (3q22.1), TF (3q22.1), TOPBP1 (3q22.1), ASTE1 (3q22.1), ATP2C1 (3q22.1), COL6A6 (3q22.1), PIK3R4 (3q22.1), ATR (3q23), CLSTN2 (3q23), PAQR9 (3q23), PLS1 (3q23), TRIM42 (3q23), TRPC1 (3q23), U2SURP (3q23), PLOD2 (3q24), ANKUB1 (3q25.1), MLF1 (3q25.1), PFN2 (3q25.1), RNF13 (3q25.1), TSC22D2 (3q25.1), ACTRT3 (3q26.2), LRRC34 (3q26.2), MYNN (3q26.2), CAND1 (12q14) | 5 | (21.7) | 13 | (56.5) | 0.0399 |
| TFCP2L1 (2q14), SP3 (2q31), LRP2 (2q31.1), OLA1 (2q31.1) | 6 | (26.1) | 14 | (60.9) | 0.0399 |
| MPHOSPH8 (13q12.11), PSPC1 (13q12.11), TPTE2 (13q12.11) | 8 | (34.8) | 16 | (69.6) | 0.0399 |
| PDS5B (13q12.3), COG6 (13q14.11), PCDH20 (13q21), CLN5 (13q21.1-q32), TDRD3 (13q21.2) | 9 | (39.1) | 17 | (73.9) | 0.0399 |
| GNG11 (7q21), BET1 (7q21.1-q22), CYP51A1 (7q21.2), LRRD1 (7q21.2), SAMD9L (7q21.2), GNGT1 (7q21.3), PON3 (7q21.3), TFPI2 (7q22), COL1A2 (7q22.1), FAM3C (7q31), WNT16 (7q31), AKR1D1 (7q32-q33), JPH1 (8q21), GDAP1 (8q21.11), E2F5 (8q21.2), LRRCC1 (8q21.2), OTUD6B (8q21.3), LRP12 (8q22.2), ABRA (8q23.1) | 10 | (43.5) | 18 | (78.3) | 0.0399 |
| MRPS17 (7p11), PSPH (7p11.2), ZNF713 (7p11.2), ZPBP (7p14.3), STARD3NL (7p14-p13), ABCB5 (7p21.1), SP8 (7p21.2), TWIST1 (7p21.2), ETV1 (7p21.3), RSBN1L (7q11.23), TMEM60 (7q11.23), PHTF2 (7q11.23-q21), FZD1 (7q21), GRM3 (7q21.1-q21.2), MTERF1 (7q21.2), SDHAF3 (7q21.3), DNAJC2 (7q22), PMPCB (7q22.1), SLC26A5 (7q22.1), PSMC2 (7q22.1-q22.3), ATXN7L1 (7q22.3), CDHR3 (7q22.3), NAMPT (7q22.3), SYPL1 (7q22.3), ANKRD7 (7q31), GPR37 (7q31), LSMEM1 (7q31.1), CAV1 (7q31.1), DOCK4 (7q31.1), IFRD1 (7q31.1), ZNF277 (7q31.1), ASZ1 (7q31.2), ST7 (7q31.2), TES (7q31.2), CAPZA2 (7q31.2-q31.3), ARF5 (7q31.3), FSCN3 (7q31.3), LRRC4 (7q31.3), SND1 (7q31.3), AASS (7q31.3), LSM8 (7q31.31), FEZF1 (7q31.32), RNF133 (7q31.32), ZNF800 (7q31.33), C7orf77 (7q31.33), POT1 (7q31.33), RNF148 (7q31.33), MKLN1 (7q32), PAX4 (7q32), CEP41 (7q32), CPA1 (7q32), CPA5 (7q32), NRF1 (7q32), UBE2H (7q32), GCC1 (7q32.1), AHCYL2 (7q32.1), SMKR1 (7q32.1), STRIP2 (7q32.1), TSPAN33 (7q32.1), KLHDC10 (7q32.2), ZC3HC1 (7q32.2), SMO (7q32.3), PODXL (7q32-q33), LUZP6 (7q33) | 11 | (47.8) | 19 | (82.6) | 0.0399 |
| POM121L12 (7p12.1), ABCA13 (7p12.3), C7orf57 (7p12.3), HUS1 (7p12.3), SUN3 (7p12.3), UPP1 (7p12.3), TBX20 (7p14.3), MPP6 (7p15), OSBPL3 (7p15), PPP1R17 (7p15), NPY (7p15.1), CHN2 (7p15.3), HDAC9 (7p21.1), ACTB (7p22), CHST12 (7p22), FSCN1 (7p22), MAD1L1 (7p22), TTYH3 (7p22), TNRC18 (7p22.1), SLC29A4 (7p22.1), WIPI2 (7p22.1), FBXL18 (7p22.2), GNA12 (7p22.2), LFNG (7p22.2), AMZ1 (7p22.3), BRAT1 (7p22.3), EIF3B (7p22.3), GRIFIN (7p22.3), IQCE (7p22.3), SNX8 (7p22.3), PON1 (7q21.3), PPP1R9A (7q21.3), SLC25A13 (7q21.3), LAMB1 (7q22), BCAP29 (7q22.3), CBLL1 (7q22.3), DUS4L (7q22-q31), COG5 (7q31), GPR85 (7q31), SLC26A3 (7q31), SLC26A4 (7q31), IMMP2L (7q31), TMEM168 (7q31.32), DLD (7q31-q32) | 12 | (52.2) | 20 | (87) | 0.0399 |
| JAZF1 (7p15.2-p15.1) | 14 | (60.9) | 22 | (95.7) | 0.0399 |
| JUN (1p32-p31), AKR1A1 (1p33-p32), CCDC17 (1p34.1), GPBP1L1 (1p34.1), MMACHC (1p34.1), NASP (1p34.1), PRDX1 (1p34.1), TMEM69 (1p34.1), EVA1B (1p34.3), STK40 (1p34.3), THRAP3 (1p34.3), SETMAR (3p26.1), SCGB3A2 (5q32), SPINK1 (5q32), ARHGDIG (16p13.3), PDIA2 (16p13.3) | 0 | (0) | 7 | (30.4) | 0.0466 |
| HAO2 (1p12), PHGDH (1p12), ZNF697 (1p12), HSD3B1 (1p13.1), HSD3B2 (1p13.1), HMGCS2 (1p13-p12), ABCD3 (1p21.3), F3 (1p22-p21), NFIA (1p31.3-p31.2), EPS15 (1p32), GPX7 (1p32), ORC1 (1p32), RNF11 (1p32), TESK2 (1p32), BTF3L4 (1p32.3), C1orf185 (1p32.3), CC2D1B (1p32.3), DMRTA2 (1p32.3), PRPF38A (1p32.3), TXNDC12 (1p32.3), USP24 (1p32.3), ZFYVE9 (1p32.3), RAB3B (1p32-p31), FAF1 (1p33), TOE1 (1p33), ELAVL4 (1p34), BEST4 (1p34.1), BTBD19 (1p34.1), EIF2B3 (1p34.1), HPDL (1p34.1), KIF2C (1p34.1), MUTYH (1p34.1), PLK3 (1p34.1), PTCH2 (1p34.1), TCTEX1D4 (1p34.1), ZSWIM5 (1p34.1), RPS8 (1p34.1-p32), COL8A2 (1p34.2), ADPRHL2 (1p34.3), C1orf94 (1p34.3), CSMD2 (1p34.3), PHC2 (1p34.3), SFPQ (1p34.3), ZMYM1 (1p34.3), ZSCAN20 (1p34.3), A3GALT2 (1p35.1), HMGB4 (1p35.1), ZNF362 (1p35.1) | 1 | (4.3) | 8 | (34.8) | 0.0466 |
| **LOH** |  |  |  |  |  |
| TSHR (14q31) | 13 | (56.5) | 3 | (13) | 0.0486 |
| **CN-LOH** |  |  |  |  |  |
| None |  |  |  |  |  |

ISL, invasive submucosal lesion; IFL, invasive front lesion; LOH, loss of heterozygosity; CN-LOH, copy neutral loss of heterozygosity.
