# Supplementary material for: Genome‐wide analysis of colorectal cancer based on gene‐based somatic copy number alterations during neoplastic progression within the same tumor
Source: Cancer Med. 2022 Aug 3;12(4):4446–54. doi: 10.1002/cam4.5117 (PMC9972084; doi:10.1002/cam4.5117)
Supplement: Supplementary file 2 — Figure S1 [file CAM4-12-4446-s001.pdf]

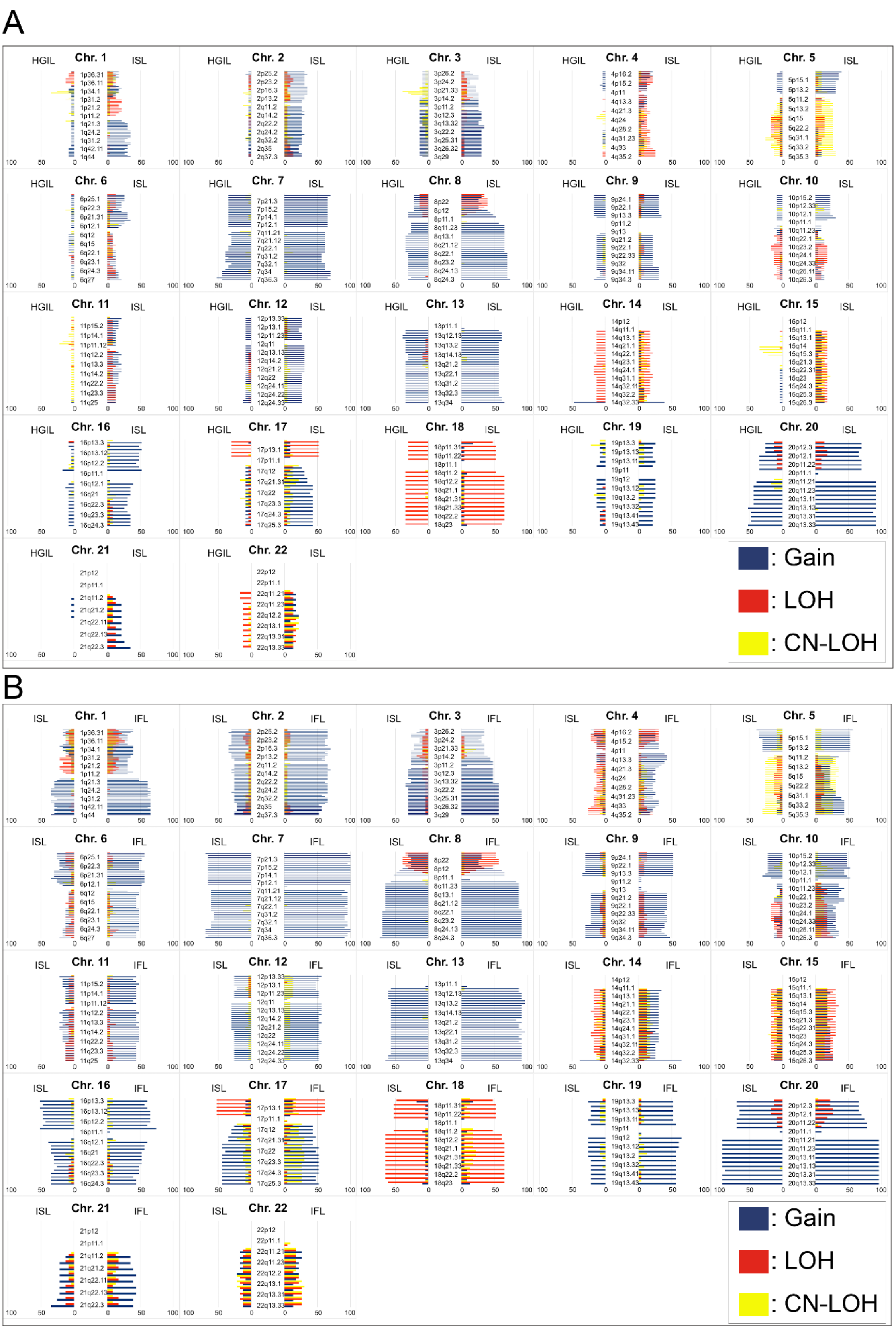

Figure S1. Ideogram of somatic copy number alterations in three lesion components (high-grade intramucosal lesion [HGIL], invasive submucosal lesion [ISL], and invasive front lesion [IFL]) within the same tumor. Chromosomes are ordered from 1 to 22. The colored horizontal lines represent the frequencies of gains, loss of heterozygosity (LOH), and copy-neutral loss of heterozygosity (CN-LOH). A. The lines on the left and right indicate SCNAs found in HGIL and ISL, respectively (blue, gain; red, LOH; yellow, CN-LOH). B. The lines on the left and right indicate SCNAs found in ISL and IFL, respectively (blue, gain; red, LOH; yellow, CN-LOH).
